# A comprehensive risk model of disulfidoptosis-related lncRNAs predicts prognosis and therapeutic implications in bladder cancer

**DOI:** 10.1016/j.bbrep.2025.102060

**Published:** 2025-05-26

**Authors:** Zhixiong Zhang, Jinghua Zhong, Muhammad Sarfaraz Iqbal, Zhiwen Zeng, Xiaolu Duan

**Affiliations:** a Department of Urology, The First Affiliated Hospital of Guangzhou Medical University, Guangdong Provincial Key Laboratory of Urology, Guangdong Engineering Research Center of Urinary Minimally Invasive Surgery Robot and Intelligent Equipment, Guangzhou Institute of Urology, China; b Department for Bipolar Disorders, Shenzhen Kangning Hospital, Shenzhen Mental Health Center, Shenzhen, 518020, China

**Keywords:** Bladder cancer, Disulfidoptosis, Long non-coding RNA, Prognostic model, Personalized treatment

## Abstract

**Background:**

Disulfidoptosis is an emerging form of regulated cell death; however, the roles of its associated long non-coding RNAs (dr-lncRNAs) in bladder cancer (BLCA) remain poorly characterized. By leveraging the most comprehensive curated dataset of disulfidoptosis-related genes to date, we systematically developed and validated a novel dr-lncRNA signature that elucidates the prognostic significance and immune microenvironmental dynamics in BLCA.

**Methods:**

The Cancer Genome Atlas (TCGA) database was utilized to extract significant clinical and RNA sequencing data of BLCA patients. Cox and Lasso regression with several variables was used to create a risk model. ROC, Kaplan-Meier, and nomogram analyses were carefully reviewed for validity. The validated study evaluated intricate interactions between functional enrichment, immune cell infiltration, cancer mutation load, and treatment sensitivity. Unsupervised consensus clustering identified subgroup patterns that reflected immune system alterations, medication susceptibility, and prognosis.

**Results:**

Nine lncRNAs significantly correlated with prognosis were collectively identified, subsequently forming the basis for constructing a risk model consisting of seven lncRNAs. The model exhibited significant superiority in predicting patient outcomes, effectively distinguishing between high-risk from low-risk individuals. Functional enrichment analysis uncovered their potential involvement in immune-related biological pathways. Patients in the high-risk group exhibited higher tumor mutation burdens, more active immune functions and a higher sensitivity to chemotherapeutic drugs. Variations among BLCA subgroups were identified by consensus cluster analysis, including clinical characteristics, prognosis, lncRNA expression, immune cell infiltration, and immune checkpoint profiles.

**Conclusion:**

The dr-lncRNAs-based risk model presents a promising tool for predicting prognosis and guiding personalized immunotherapy and treatment strategies in BLCA patients.

## Highlights

• Identified 7 disulfidoptosis-related lncRNAs predictive of prognosis in BLCA.
• Developed a robust risk model surpassing traditional factors in survival prediction.
• High-risk patients exhibit increased tumor mutation burden and immune activity.
• Consensus clustering identifies subgroups with distinct clinical and immune traits.

## 1 Introduction

Bladder cancer (BLCA) is a prevalent malignant neoplasm of the urinary system, with an annual global incidence exceeding 550,000 new cases. Approximately 25 % of cases are classified as muscle-invasive BLCA, contributing to an annual mortality rate surpassing 200,000 [1,2]. Despite advances in therapeutic interventions, over 50 % of patients experience relapse within five years of surgery, and the prognosis remains poor due to the high rate of metastasis. The 5-year survival rate for BLCA is significantly reduced by the tendency of cancer cells to spread to distant organs, which underscores the urgent need for novel therapeutic strategies[[3], [4], [5]].

Disulfidoptosis is a recently identified form of cell death characterized by the abnormal accumulation of intracellular disulfides in glucose-deprived conditions, primarily in cells with elevated SLC7A11 expression. This process is driven by the atypical formation of disulfide bonds between cytoskeletal proteins, leading to cytoskeletal disintegration, cellular shrinkage, and eventual cell death. Interestingly, while SLC7A11 upregulation promotes tumor development, it also renders metastatic cancer cells more susceptible to oxidative stress, thus impeding tumor spread. This dual effect highlights a previously unrecognized mechanism of cellular death and provides valuable insights into tumor biology and metastasis regulation [6,7].

LncRNAs have been recognized as crucial regulators in the initiation and progression of BLCA, influencing key processes such as cell proliferation, apoptosis evasion, migration, invasion, and alterations in metabolic pathways [8,9]. They exert their effects through various mechanisms, including acting as microRNA decoys, regulating transcription, and modulating critical signaling networks [10,11]. Additionally, lncRNAs play a significant role in shaping the BLCA microenvironment, influencing interactions between tumor cells, surrounding stromal tissue, and immune cells [12]. However, despite their recognized importance, the roles of lncRNAs associated with disulfidoptosis in BLCA remain poorly understood, and their prognostic significance remains largely unexplored. Notably, although the molecular heterogeneity of BLCA subtypes is well-established, a systematic characterization of dr-lncRNA signatures across clinically distinct BLCA subgroups remains lacking. This knowledge gap persists despite accumulating evidence implicating subtype-specific lncRNA regulatory networks in oncogenic processes [13]. Consequently, delineating the prognostic utility of dr-lncRNAs is imperative for identifying novel biomarkers and advancing personalized therapeutic strategies in BLCA.

Here, we constructed a prognostic risk model based on seven dr-lncRNAs, demonstrating strong predictive accuracy in distinguishing high-risk from low-risk bladder cancer patients. By uncovering and validating dr-lncRNAs, this work expands our understanding of BLCA and provides new perspectives on personalized treatment approaches and prognostic markers.

## 2 Materials and methods

### 2.1 Public data collection

**Supplementary Fig. S1:**
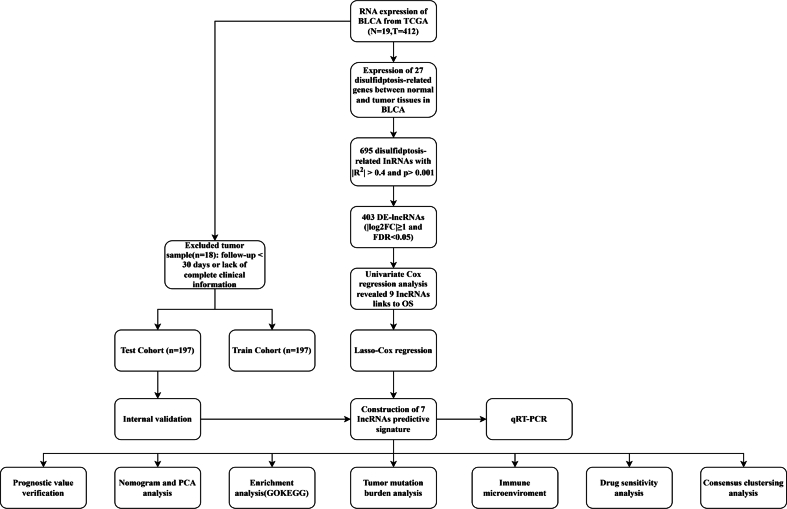
Study Flowchart. Abbreviations: BLCA, Bladder urothelial carcinoma; TCGA, The Cancer Genome Atlas; lncRNAs, long noncoding RNAs; OS, overall survival; LASSO, least absolute shrinkage and selection operator; PCA, principal component analysis; GO, Gene Ontology; KEGG, Kyoto Encyclopedia of Genes and Genomes

RNA-seq transcriptomic data and clinical information for BLCA were obtained from patients in the TCGA database (https://tcga-data.nci.nih.gov/tcga/). To maintain data reliability, we excluded patients with incomplete overall survival (OS) information or OS duration of less than 30 days. This process yielded a dataset consisting of RNA-seq data from 394 BLCA tumor samples, 19 normal tissue samples, and mutation data from BLCA patients in MAF format from TCGA (Supplementary Fig. S1).

### 2.2 Screening and differential expression analysis of dr-lncRNAs

The study initially identified 27 genes associated with disulfidoptosis (Supplementary Table S1) and constructed an mRNA-lncRNA co-expression network using a Pearson correlation coefficient threshold of greater than 0.4 (p < 0.001) [6]. The network's structure was accurately depicted with a Sankey diagram. A stringent selection process identified DE-lncRNAs using the “limma” R package with criteria of false discovery rate (FDR) less than 0.05 and absolute log2 fold change (|log2FC|) greater than 1. Expression patterns of these lncRNAs were visualized using volcano plots. lncRNAs with significant predictive value (P < 0.01) were identified through univariate COX regression analysis.

### 2.3 Establishment and validation of dr-lncRNAs signature model in BLCA

The TCGA dataset was meticulously partitioned into training and test sets in an equal 1:1 ratio. A comprehensive prognostic model for dr-lncRNAs was constructed using LASSO regression and multivariate COX regression analyses. The risk score formula, Risk score = Σ(Coef ∗ EXP), incorporated regression coefficients (Coef) estimated through multivariate COX regression analysis and expression levels (EXP) of the lncRNAs with prognostic significance. The autonomous prognostic power of the risk prediction model was evaluated using univariate and multivariate COX analyses [14]. ROC curves were computed for 1-year, 3-year, and 5-year survival, and the model's performance was assessed by area under curve (AUC) values [15]. According to established guidelines, AUC values between 0.7 and 0.8 indicate fair discrimination, 0.8 to 0.9 represent good discrimination, and values above 0.9 are considered excellent [16]. Correlation studies explored relationships between risk scores and clinical-pathological characteristics. The prognostic efficacy of the model was evaluated through Kaplan-Meier (K-M) survival curve analysis, specifically examining its performance in predicting outcomes for BLCA patients with varying clinical and pathological characteristics.

### 2.4 Establishment of nomogram and principle component analysis (PCA)

The current study has developed a comprehensive nomogram that integrates crucial clinical characteristics, including age, gender, T stage, M stage, N stage, clinical stage, histological grade, and associated risk scores. The calibration curves were carefully examined to identify any discrepancies between the expected and observed results, while the nomogram accurately estimated the likelihood of survival over different time periods, specifically 1, 3, and 5 years. The assessment of the model's precision was based on the utilization of the Concordance Index (C-index), a reliable statistic for predicting survival rates among patients with BLCA. The application of PCA allowed for the identification of inherent gene-expression patterns within distinct gene sets in individuals with BLCA. This analysis facilitated the visualization of the distribution of high-risk and low-risk groups across various gene sets, thereby enhancing the comprehensiveness of our research efforts.

### 2.5 Functional enrichment analysis

Differential gene expression analysis was conducted using the “limma” R package to identify genes exhibiting differential expression across high-risk and low-risk groups. This analysis was guided by rigorous criteria, requiring a minimum |log2FC| of 1 and an FDR below 0.05. To boost interpretability, the “clusterProfiler” R package was utilized to graphically depict the results of Gene Ontology (GO) and Kyoto Encyclopedia of Genes and Genomes (KEGG) enrichment studies.

### 2.6 Tumor immune microenvironment of risk prognostic signature

The study employed Spearman's correlation analysis to investigate the associations among immune cell subtypes, risk ratings, and immune checkpoint expression. The study employed single-sample gene set enrichment analysis (ssGSEA) to measure the scores of invading immune cells and evaluate the activation of immune-related pathways. The ESTIMATE algorithm was utilized to calculate stromal and immunological scores, enabling the investigation of the relationship between risk scores and the expression of immune checkpoints.

### 2.7 The association between tumor mutation burden and immune infiltration with survival analysis

The tumor mutation burden (TMB) was calculated using the “maftools” R package by performing a comprehensive analysis of somatic mutation profiles in high-risk and low-risk groups. Simultaneously, Tumor Immune Dysfunction and Exclusion (TIDE) analysis (http://tide.dfci.harvard.edu/) was systematically applied to quantify tumor immune evasion mechanisms. The TIDE algorithm integrates two primary mechanisms of tumor immune evasion: 1) T-cell dysfunction, which assesses the likelihood of tumor-infiltrating cytotoxic T lymphocytes (CTLs) losing their anti-tumor activity, and 2) T-cell exclusion, which evaluates the suppression of CTL infiltration into the tumor microenvironment. The TIDE score inversely correlates with immunotherapy efficacy, where higher scores indicate greater immune evasion and poorer predicted response to immune checkpoint inhibitors. Input data included standardized gene expression profiles of BLCA patients from the TCGA cohort. Default parameters were applied for signature gene sets and statistical thresholds as defined by the TIDE developers. Additionally, TIMER analysis was conducted to evaluate immune cell infiltration patterns associated with high-mutation-frequency genes. Immunotherapy response predictions were generated using the “pRRophetic” R package, with statistical validation via Wilcoxon signed-rank tests.

### 2.8 Immunotherapy for risk signature and prediction of potential drugs

In order to evaluate the response to drug treatments in BLCA, we employed the “pRRophetic” R package for predicting the sensitivity of both high-risk and low-risk BLCA patients to commonly used drugs, quantified by the half-maximal inhibitory concentration (IC50) [17]. Differences between these groups were assessed utilizing the Wilcoxon signed-rank test. Furthermore, we investigated the relationship between drug sensitivity and risk scores by means of spearman correlation analysis.

### 2.9 Consensus cluster analysis and immunological correlation

Additionally, consensus cluster analysis was conducted using the “ConsensusClusterPlus” algorithm to group BLCA samples into three unique subtypes. Within each subtype, we conducted subsequent K-M survival analyses, created heatmaps, and assessed the state of immune infiltration and the expression of immune checkpoint genes.

### 2.10 Cell culture

BLCA lines (5637, T24, J82, and EJ1) and the normal human bladder epithelial line SV-HUC-1 were acquired from the Chinese Academy of Science in Shanghai. Cultivation of SV-HUC-1 cells occurred in F12 medium (Gibco), while J82 and EJ1 were cultured in DMEM (Gibco), and T24 and 5637 cell lines were sustained in RPMI-1640 (Gibco). All culture media were supplemented with 10 % fetal bovine serum (PAN). Incubation of cells was carried out at 37 °C with 5 % CO2. Subculturing was performed at 70–80 % confluence using trypsin-EDTA (Gibco). Cell passages were conducted during the logarithmic growth phase for all experiments.

### 2.11 Quantitative real-time PCR(qRT-PCR)

Total RNA was extracted from the cell lines using TRIzol reagent (Vazyme). cDNA synthesis was performed using 1 μg of total RNA with the PrimeScript RT Reagent Kit (TaKaRa) following the manufacturer's instructions. Real-time PCR was conducted on a real-time PCR system (Roche) utilizing TB green (TaKaRa) as the fluorescent dye. Specific primers for target genes are detailed in Supplementary Table S2, and GAPDH served as the normalization control.

### 2.12 Statistical analysis

Statistical analyses were performed using R software (version 4.2.3). Pearson correlation coefficients were used to construct the mRNA-lncRNA co-expression network, while Spearman correlation was employed for other correlation analyses. Wilcoxon rank-sum tests were used to examine differences in mRNA and lncRNA expression levels. Kaplan-Meier survival analysis was performed using log-rank tests. The predictive relevance of the risk score and clinical features was assessed using both univariate and multivariate Cox regression analyses, with statistical significance set at p < 0.05.

## 3 Results

### 3.1 Identification of disulfidoptosis-related lncRNAs (Dr-lncRNAs)

**Fig. 1:**
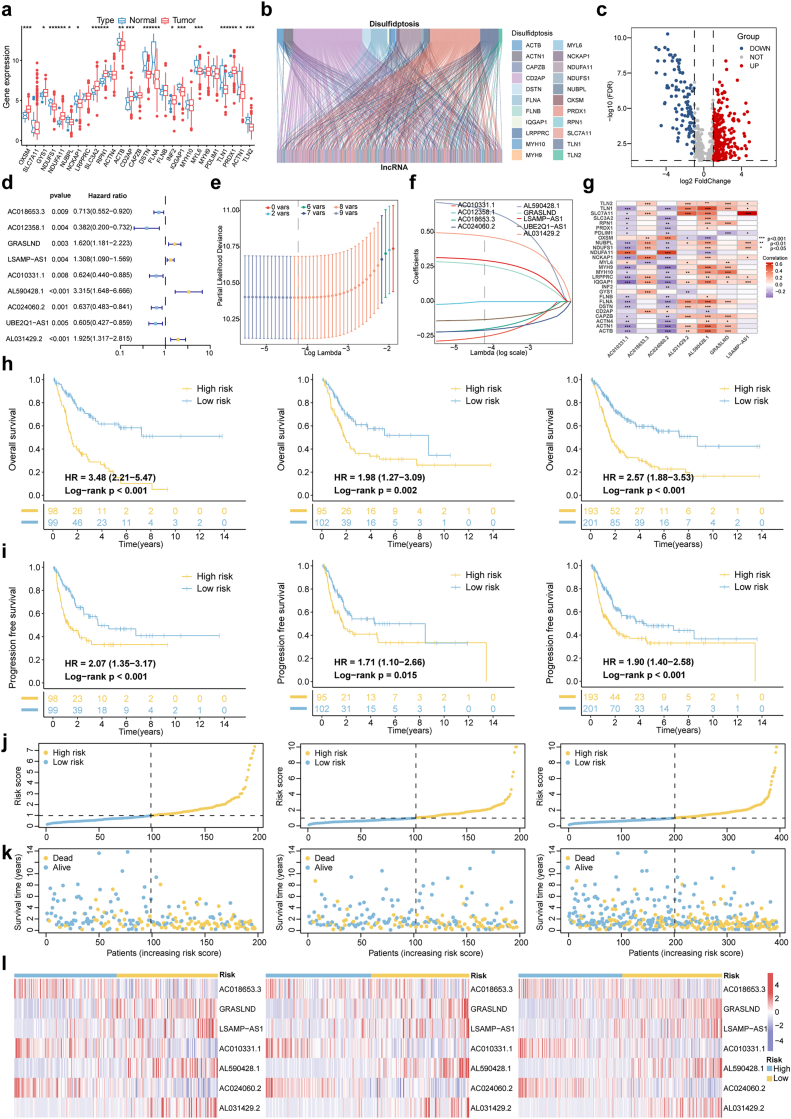
Development and evaluation of predictive models for dr-lncRNAs in BLCA. **(a)** Differential expression of 19 DRGs between BLCA and normal tissues. **(b)** Sankey diagram showing relationships between 27 DRGs and 695 dr-lncRNAs. **(c)** Volcano plot of differentially expressed lncRNAs, with 301 upregulated and 102 downregulated genes. **(d)** Univariate Cox regression identified 9 prognostic dr-lncRNAs. **(e)** 10-fold cross-validation for variable selection in LASSO regression (training cohort). **(f)** LASSO coefficients distribution for dr-lncRNAs. **(g)** Correlation between 27 DRGs and 9 prognostic dr-lncRNAs. **(h)** Kaplan-Meier OS curves for high- and low-risk groups in the training, test, and entire cohorts. **(i)** Kaplan-Meier PFS curves for high- and low-risk groups in the training, test, and entire cohorts. **(j)** Risk class distribution in the training, test, and entire cohorts. **(k)** Survival status of patients in the training, test, and entire cohorts. **(l)** Expression of prognostic dr-lncRNAs in the training, test, and entire cohorts. dr-lncRNAs, disulfidoptosis-related long noncoding RNAs; DRGs, disulfidoptosis-related genes; ∗P < 0.05, ∗∗P < 0.01, ∗∗∗P < 0.001.

Initially, the expression patterns of genes associated with disulfidoptosis were analyzed in patients diagnosed with BLCA. A curated set of 27 disulfidoptosis-associated genes was examined to compare their expression profiles in healthy individuals and those with BLCA. Notably, 19 of these genes, including SLC3A2, FLNA, and MYH9, showed significant changes in expression levels (Fig. 1a). Using the expression profiles of these 27 genes, 695 co-expressed lncRNAs were identified (Fig. 1b). The selection of these lncRNAs was based on strict criteria, requiring a correlation coefficient >0.4 and a p-value <0.001 (Supplementary Table S3). Differential expression analysis of tumor samples revealed 301 upregulated and 102 downregulated lncRNAs, illustrated in the volcano plot (Fig. 1c). Further, univariate Cox regression analysis identified 9 lncRNAs with significant prognostic value (Fig. 1d).

### 3.2 TCGA cohort risk signature model construction

Data from 412 BLCA patients were retrieved from the TCGA database, including survival and clinical information. After excluding cases with incomplete clinical data, 394 full cases were included in the analysis. Clinical-pathological markers showed no statistically significant differences following a 1:1 random split into training and testing sets (Table 1). A risk signature model comprising 7 unique lncRNAs was constructed using LASSO and multivariate Cox regression analysis (Fig. 1e–f). The risk score was calculated using the following formula: Risk score = (−0.286 ∗ AC018653.3 exp.) + (0.304 ∗ GRASLND exp.) + (0.328 ∗ LSAMP-AS1 exp.) + (−0.294 ∗ AC010331.1 exp.) + (0.723 ∗ AL590428.1 exp.) + (0.521 ∗ AL031429.2 exp.). A correlation heatmap was generated to illustrate the associations between disulfidoptosis-related genes and these 7 lncRNAs (Fig. 1g).

**Table 1:** Clinical characteristics and statistical comparison of BLCA patients in training and test cohorts.

Covariates	Type	Total	Test	Train	P-value
Age	≤65	158(40.1 %)	79(40.1 %)	79(40.1 %)	1
	>65	236(59.9 %)	118(59.9 %)	118(59.9 %)	
Gender	Female	103(26.14 %)	55(27.92 %)	48(24.37 %)	0.4915
	Male	291(73.86 %)	142(72.08 %)	149(75.63 %)	
Grade	High Grade	373(94.67 %)	187(94.92 %)	186(94.42 %)	1
	Low Grade	18(4.57 %)	9(4.57 %)	9(4.57 %)	
	unknow	3(0.76 %)	1(0.51 %)	2(1.02 %)	
Stage	Stage I	2(0.51 %)	1(0.51 %)	1(0.51 %)	0.9862
	Stage II	123(31.22 %)	60(30.46 %)	63(31.98 %)	
	Stage III	138(35.03 %)	69(35.03 %)	69(35.03 %)	
	Stage IV	129(32.74 %)	66(33.5 %)	63(31.98 %)	
	unknow	2(0.51 %)	1(0.51 %)	1(0.51 %)	
T	T0	1(0.25 %)	0(0 %)	1(0.51 %)	0.6648
	T1	3(0.76 %)	2(1.02 %)	1(0.51 %)	
	T2	112(28.43 %)	62(31.47 %)	50(25.38 %)	
	T3	190(48.22 %)	94(47.72 %)	96(48.73 %)	
	T4	56(14.21 %)	30(15.23 %)	26(13.2 %)	
	unknow	32(8.12 %)	9(4.57 %)	23(11.68 %)	
M	M0	188(47.72 %)	79(40.1 %)	109(55.33 %)	0.4287
	M1	10(2.54 %)	6(3.05 %)	4(2.03 %)	
	unknow	196(49.75 %)	112(56.85 %)	84(42.64 %)	
N	N0	228(57.87 %)	116(58.88 %)	112(56.85 %)	0.8137
	N1	44(11.17 %)	20(10.15 %)	24(12.18 %)	
	N2	75(19.04 %)	41(20.81 %)	34(17.26 %)	
	N3	6(1.52 %)	3(1.52 %)	3(1.52 %)	
	unknow	41(10.41 %)	17(8.63 %)	24(12.18 %)	

The clinical characteristics of BLCA patients in the training and test cohorts.

### 3.3 The correlation of survival status and genetic signature

By stratifying the entire cohort, as well as the training and test sets, into high-risk and low-risk groups based on the median risk score, we obtained critical insights into the predictive performance of the proposed model. Kaplan–Meier analysis revealed that patients in the high-risk groups exhibited significantly reduced OS and progression-free survival (PFS) compared to those in the low-risk groups (Fig. 1h and i), underscoring the model's strong prognostic value. Consistent trends across all subsets further reinforced the robustness and generalizability of the risk score (Fig. 1j and k). Heatmap analysis of the seven lncRNAs included in the model revealed distinct expression patterns between the risk groups (Fig. 1l). Notably, GRASLND, LSAMP-AS1, AL590428.1, and AL031429.2 were upregulated in the high-risk group, whereas AC018653.3, AC010331.1, and AC024060.2 were enriched in the low-risk group. These consistent trends highlight substantial molecular differences between risk categories and further support the robustness and clinical applicability of the risk model.

### 3.4 Assessment of the prognostic value of risk models

**Fig. 2:**
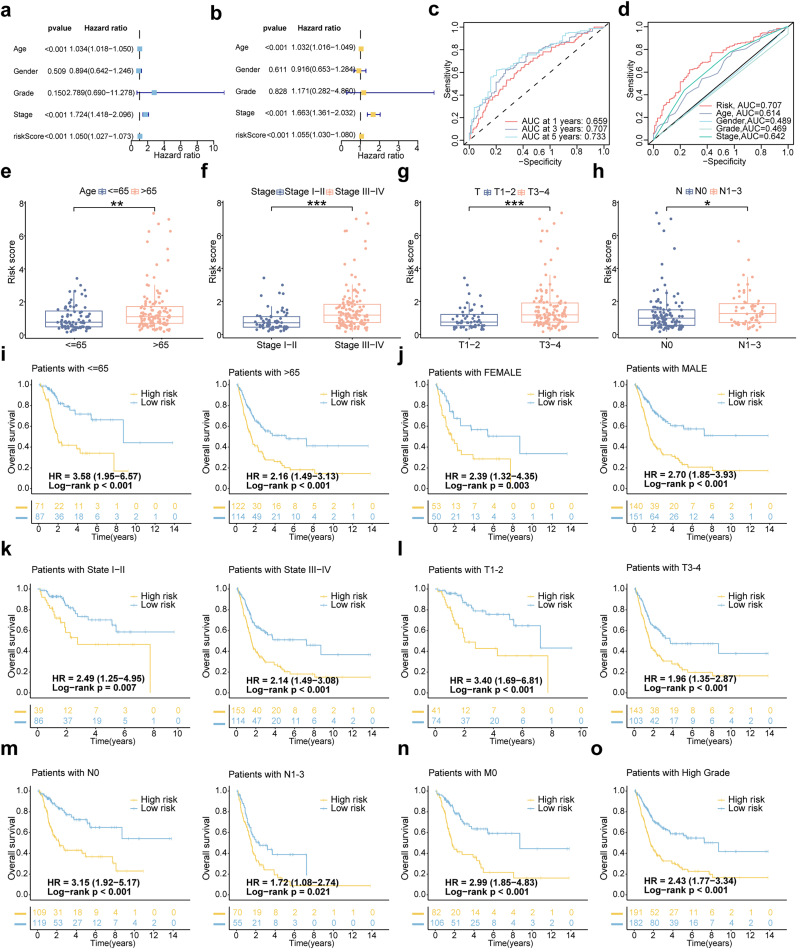
Clinicopathological correlations and independent prognostic factors of the dr-lncRNA signature. **(a)** Univariate and **(b)** multivariate independent prognostic analysis of risk score and clinical variables. **(c)** ROC curves for the risk signature's 1-, 3-, and 5-year survival rates. **(d)** ROC curves for risk score and clinical characteristics. Risk score distribution in patients with different clinicopathological features: age **(e)**, AJCC stage **(f)**, T stage **(g)**, and N stage **(h)**. Kaplan-Meier survival analysis for high- and low-risk groups based on age **(i)**, gender **(j)**, AJCC stage **(k)**, T stage **(l)**, N stage **(m)**, M stage **(n)**, and histological grade **(o)**. ROC, receiver operating characteristic; T, tumor; N, lymph node; M, metastasis; ∗P < 0.05, ∗∗P < 0.01, ∗∗∗P < 0.001.

To assess the risk model's potential as an independent prognostic factor for BLCA, univariate and multivariate Cox regression analyses were performed. The univariate analysis identified significant associations between age, stage, risk score, and OS (Fig. 2a). In the multivariate analysis, age (HR = 1.032, 95 % CI: 1.016–1.049, p < 0.001), stage (HR = 1.663, 95 % CI: 1.361–2.032, p < 0.001), and risk score (HR = 1.055, 95 % CI: 1.030–1.080, p < 0.001) were independent predictors of OS (Fig. 2b). These findings underscore the critical role of the risk score as a significant independent prognostic factor intricately linked to the survival outcomes of individuals diagnosed with BLCA. Furthermore, the predictive performance of the model was evaluated using time-dependent ROC curve analyses. The model yielded AUC values of 0.659, 0.707, and 0.733 for 1-, 3-, and 5-year OS, respectively (Fig. 2c). According to the established benchmarks, AUC values between 0.7 and 0.8 are indicative of fair to good discriminatory ability, supporting the model's clinical relevance in intermediate- and long-term survival prediction. Comparative ROC analysis further confirmed that the risk score (AUC = 0.707) outperformed traditional clinical features such as age (0.614), gender (0.489), stage (0.469), and grade (0.642) (Fig. 2d). These results provide strong evidence that our dr-lncRNA-based risk score offers superior prognostic accuracy to conventional clinical parameters.

### 3.5 Assessing the correlation between risk scores and clinical pathological parameters

To evaluate the predictive value of the risk model in BLCA patients, a correlation analysis was performed to assess the relationship between the risk score and clinical-pathological parameters. Survival curves were compared across high-risk and low-risk groups, stratified by various clinical characteristics. The results showed that older patients, those with advanced stage cancer (T3-4), and those with lymph node metastases had higher risk scores (Fig. 2e–h). Additionally, the risk score effectively predicted patient outcomes based on key clinical-pathological variables (Fig. 2i–o).

### 3.6 Construction and validation of the OS nomogram and principal component analysis

**Fig. 3:**
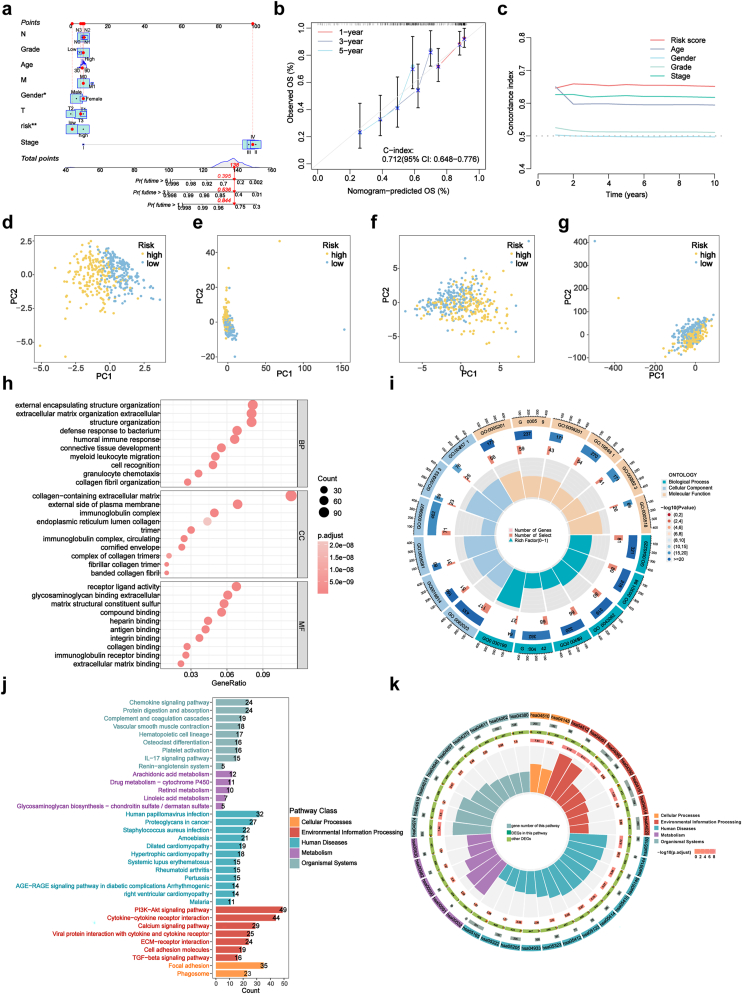
Creation and validation of a nomogram and enrichment analysis of DEGs in low- and high-risk groups. **(a)** Nomogram integrating clinicopathological factors and risk scores to predict 1-, 3-, and 5-year survival probabilities for BLCA patients. **(b)** Calibration curves comparing observed and predicted overall survival at 1, 3, and 5 years. **(c)** C-index curves evaluating the discriminative ability of risk scores and clinical factors at different time points. **(d**–**g)** PCA scatterplots showing sample distribution differences between low- and high-risk groups using lncRNA profiles, dr-lncRNAs, DRGs, and the full gene expression profile. **(h, i)** GO analysis of biological processes (BP), cellular components (CC), and molecular functions (MF). **(j, k)** KEGG pathway analysis of significantly enriched pathways. DEGs, differentially expressed genes; GO, gene ontology; KEGG, Kyoto Encyclopedia of Genes and Genomes.

Our study on BLCA integrated the OS nomogram and PCA to enhance predictive accuracy. Nomograms were developed to predict 1-year, 3-year, and 5-year OS by combining clinical factors with the risk score (Fig. 3a). The nomogram demonstrated strong agreement between predicted and observed outcomes, as evidenced by calibration curves and a C-index of 0.712 (95 % CI = 0.648–0.776) (Fig. 3b), indicating favorable model performance. Moreover, the risk score outperformed traditional clinical indicators such as age, gender, grade, and stage in predicting patient outcomes (Fig. 3c). To further evaluate the discriminative power of the risk model, we performed principal component analysis (PCA), which revealed distinct transcriptomic profiles between high-risk and low-risk groups (Fig. 3d–g). These findings collectively highlight the potential of our model to stratify patients not only by prognosis but also by underlying molecular characteristics.

### 3.7 Functional enrichment analyses

Differential genes between high-risk and low-risk groups were analyzed using GO and KEGG analyses. GO analysis revealed that these genes are involved in signal transduction, cellular components, and immune-related functions such as humoral immune response, myeloid leukocyte migration, and granulocyte chemotaxis (Fig. 3h and i). KEGG analysis identified associations with immune-related pathways, including the Chemokine Signaling, IL-17 Signaling, PI3K-AKT Signaling, Cytokine-Cytokine Receptor Interaction, and Viral Protein Interaction with Cytokines and Receptors (Fig. 3j and k).

### 3.8 Tumor immune microenvironment for risk signature

**Fig. 4:**
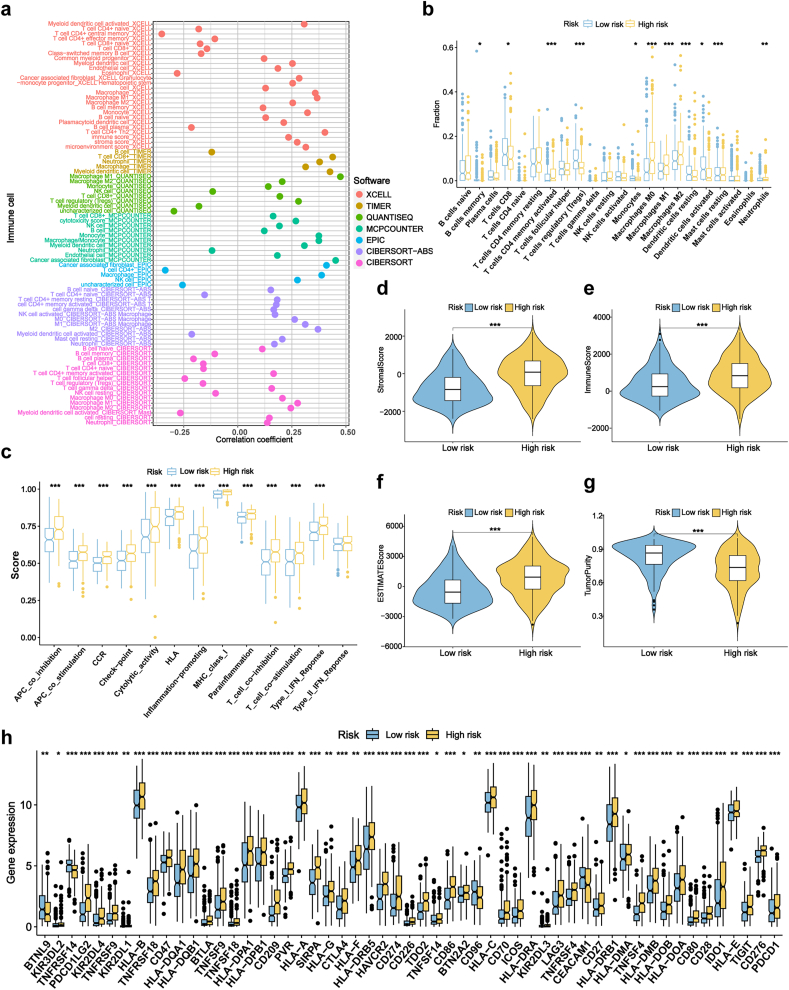
Comprehensive evaluation of the risk signature: immune cells, functions, TME, and checkpoints. **(a)** Correlation between immune cell abundance and risk scores across 7 algorithms. **(b)** Immune cell infiltration differences between low- and high-risk groups. **(c)** Differences in immune-related pathways between risk groups. **(d**–**g)** ESTIMATE analysis of immune score, stromal score, ESTIMATE score, and tumor purity between the two risk groups. **(h)** Comparison of immune checkpoint activation across risk groups. TME, tumor immune microenvironment. ∗P < 0.05, ∗∗P < 0.01, ∗∗∗P < 0.001.

**Supplementary Fig. S2:**
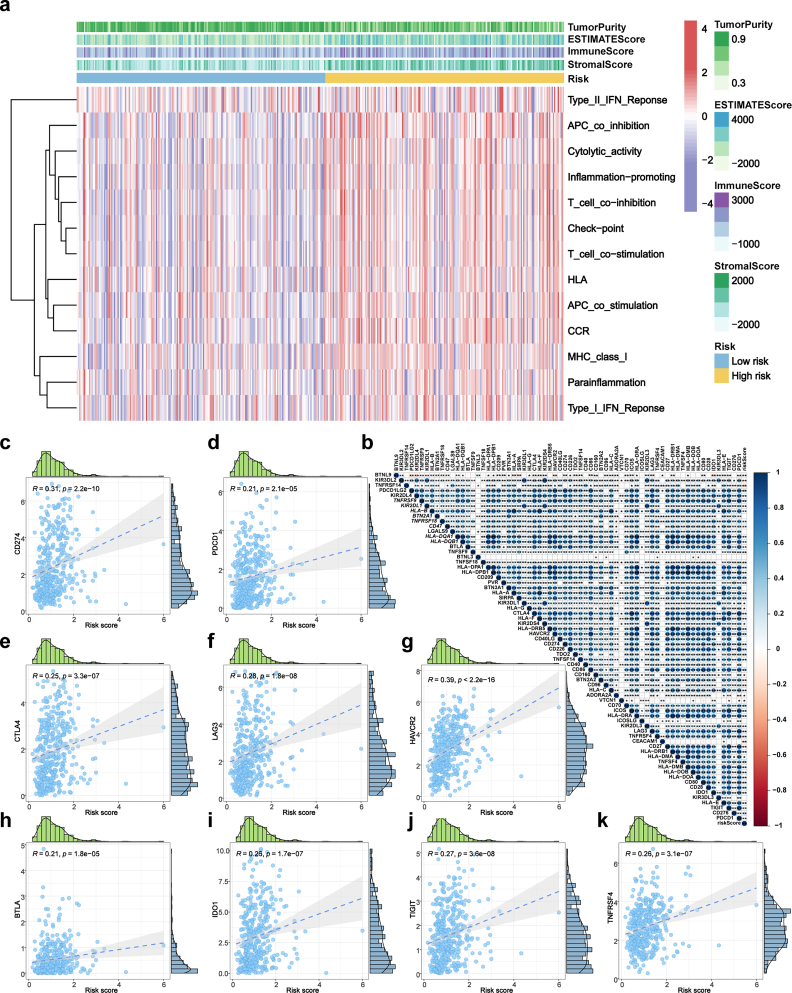
Immune-related Functions, TME, and Immune Checkpoints Analysis for the Risk Signature. (a) Heatmap showing enrichment of 13 immune functions and 4 TMEs in the two risk groups. (b) Association between immune checkpoints and risk scores. (c-k) Correlation between risk scores and immune checkpoints (CD274 (PD-L1), PDCD1 (PD-1), CTLA-4, LAG-3, HAVCR2 (TIM-3), BTLA, IDO1, TIGIT, TNFRSF4). ∗P < 0.05, ∗∗P < 0.01, ∗∗∗P < 0.001

Based on the results of the GO and KEGG enrichment analyses, we conducted an in-depth exploration of the potential connections between the risk model and the tumor immune microenvironment. Seven algorithms were used to assess the correlation between risk scores and immune cells, revealing a strong positive correlation (Fig. 4a). Quantitative assessment using ssGSEA confirmed significant differences in immune cell infiltration between the two groups, particularly in memory B cells, CD8^+^ T cells, activated CD4^+^ memory T cells, regulatory T cells, monocytes, M0/M1/M2 macrophages, dendritic cells (resting and activated), and neutrophils (Fig. 4b). Distinct activation patterns of immune-related pathways were also observed across the two risk groups. Further, ESTIMATE analysis revealed that the high-risk group exhibited significantly higher stromal and immune scores, accompanied by reduced tumor purity, suggesting a more complex and immune-enriched tumor microenvironment (Fig. 4d–g). Meanwhile, a heatmap was generated for these immune environment-related results (Supplementary Fig. S2a). Additionally, a comparative analysis of the differential expressions of immune checkpoint genes between the two risk subgroups was conducted. The results showed that immune checkpoint gene expression was significantly upregulated in the high-risk group, except for BTNL9, TNFRSF14, CD96, and CEACAM1 (Fig. 4h). These genes, including PD-1, PD-L1, CTLA-4, LAG-3, TIM-3, BTLA, IDO1, TIGIT, and TNFRSF4, showed a strong correlation with the risk score (Supplementary Fig. S2b, S2c-k).

### 3.9 Tumor mutation burden and survival analysis

**Fig. 5:**
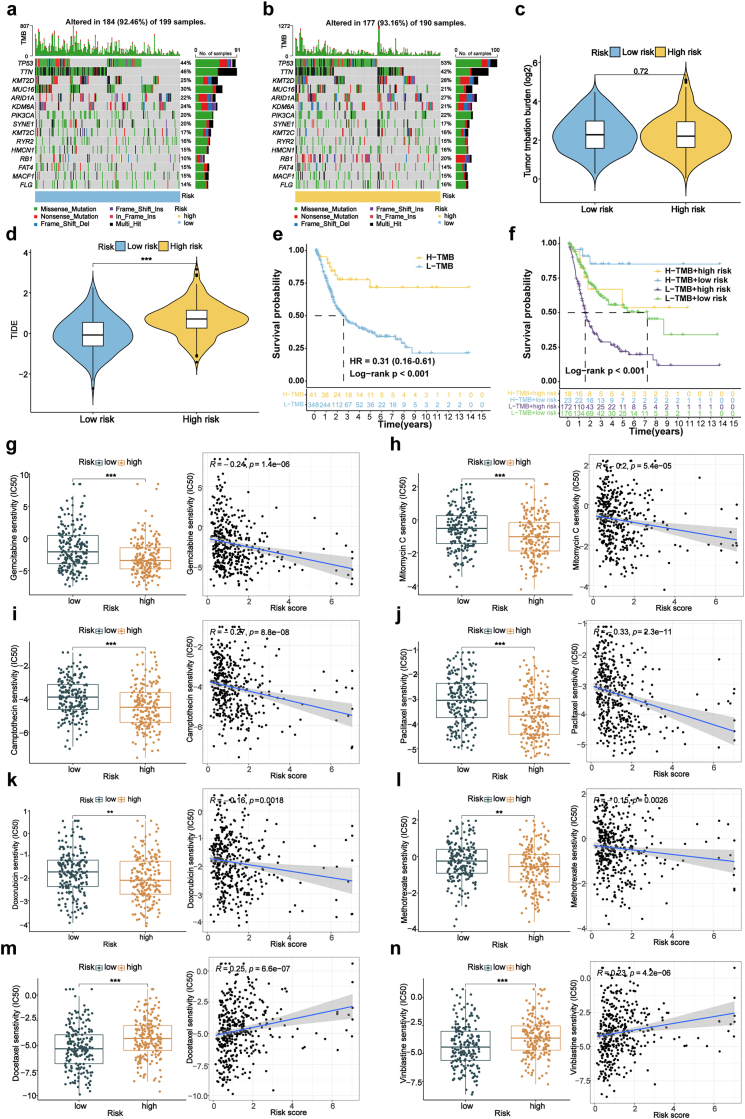
Somatic mutation landscape and drug sensitivity analysis. **(a, b)** Waterfall plots showing the top 15 genes with high mutation frequency differences between low- and high-risk groups. **(c, d)** TMB differences and TIDE prediction scores. **(e)** Kaplan-Meier curves for low- and high-TMB groups. **(f)** Kaplan-Meier curves for patients categorized by TMB and risk scores. **(g**–**n)** IC50 values and correlation of risk scores with IC50 of gemcitabine, mitomycin C, camptothecin, paclitaxel, doxorubicin, methotrexate, docetaxel, and vinblastine. TMB, tumor mutation burden; TIDE, tumor immune dysfunction and exclusion; IC50, half maximal inhibitory concentration. ∗P < 0.05, ∗∗P < 0.01, ∗∗∗P < 0.001.

**Supplementary Fig. S3:**
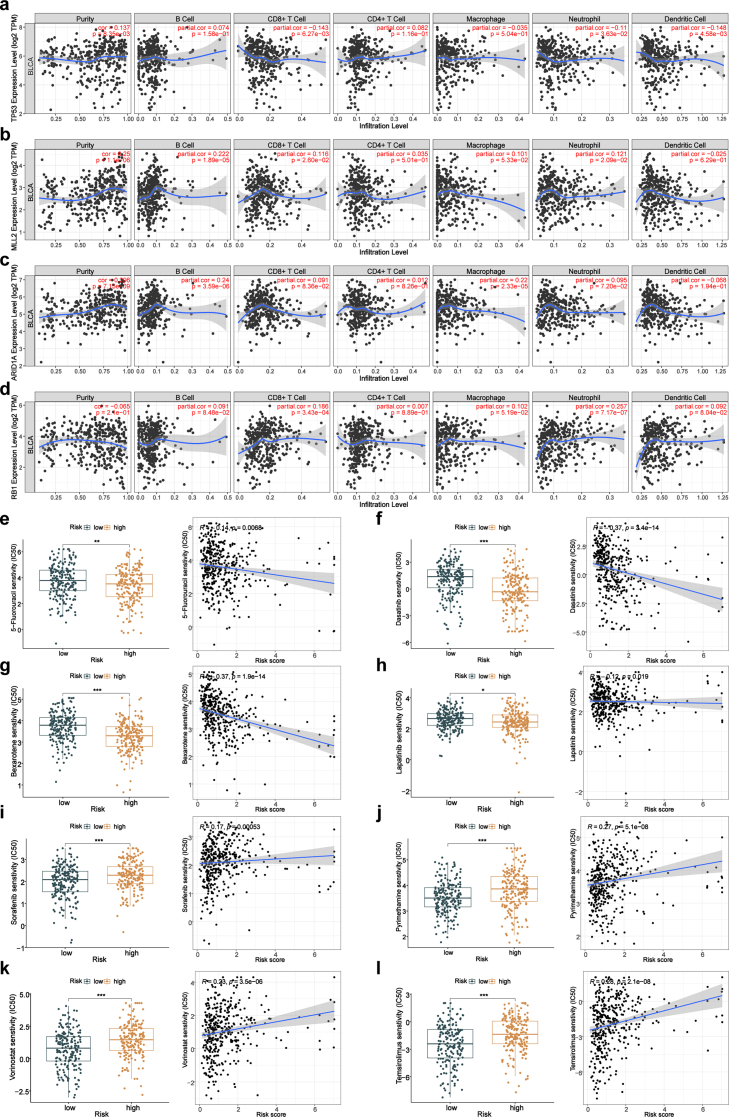
Correlation between Mutated Genes, Immune Cell Infiltration, and Drug Sensitivity. (a-d) Correlation between expression of TP53 (a), KMT2D (MLL2) (b), ARAD1A (c), and RB1 (d) with immune cell abundance in BLCA. (e-l) IC50 values and correlation between risk scores and IC50 of 5-fluorouracil, dasatinib, bexarotene, lapatinib, sorafenib, pyrimethamine, vorinostat, and temsirolimus. ∗P < 0.05, ∗∗P < 0.01, ∗∗∗P < 0.001.

The tumor mutation burden (TMB) landscape was first examined to assess the genomic alterations associated with the risk model. Waterfall plots revealed a higher frequency of somatic mutations in the high-risk subgroup, with TP53, TTN, and KMT2D among the most frequently mutated genes (Fig. 5a–c). Despite the association between elevated TMB and prolonged overall survival, as demonstrated by Kaplan-Meier analysis (Fig. 5e), patients in the high-risk group with high TMB still exhibited inferior outcomes compared to low-risk counterparts, suggesting that the risk model retains prognostic value independent of mutational burden (Fig. 5f). To further elucidate the relationship between genomic instability and immune evasion, we conducted TIDE analysis. High-risk patients showed markedly elevated TIDE scores, indicating enhanced immune escape potential and a reduced likelihood of response to immune checkpoint blockade (Fig. 5d). This was accompanied by increased expression of several key immune checkpoint genes, such as PDCD1, CD274, and CTLA4, highlighting a suppressive immune microenvironment (Supplementary Fig. S3a–d). These findings suggest that high-risk patients may be less likely to benefit from current immunotherapies, despite exhibiting high mutational loads. Collectively, the integration of TMB and TIDE metrics underscores the clinical relevance of the risk model in capturing both genomic and immunological features that influence patient prognosis and treatment response.

### 3.10 Drug sensitivity analysis based on risk signature

In this study, we analyzed medication sensitivity in BLCA using the “pRRophetic” R package to determine the IC50 values of commonly prescribed drugs and investigate correlations with risk ratings. The results revealed significant differences in drug sensitivity between risk groups. High-risk patients exhibited lower IC50 values for drugs like Gemcitabine, Mitomycin C, Paclitaxel, and Doxorubicin, indicating higher susceptibility to these drugs (Fig. 5g–l, Supplementary Fig. 3e–h). Conversely, the low-risk group showed lower IC50 values for drugs like Docetaxel, Vinblastine, Sorafenib, Pyrimethamine and Vorinostat (Fig. 5m–n, Supplementary Fig. 3i–l). These findings underscore the utility of the risk model in guiding personalized treatment strategies, tailored to the varying drug sensitivities of BLCA patients across risk profiles.

### 3.11 Analyzing the survival prospects and immunotherapy potential of different BLCA subgroups

**Fig. 6:**
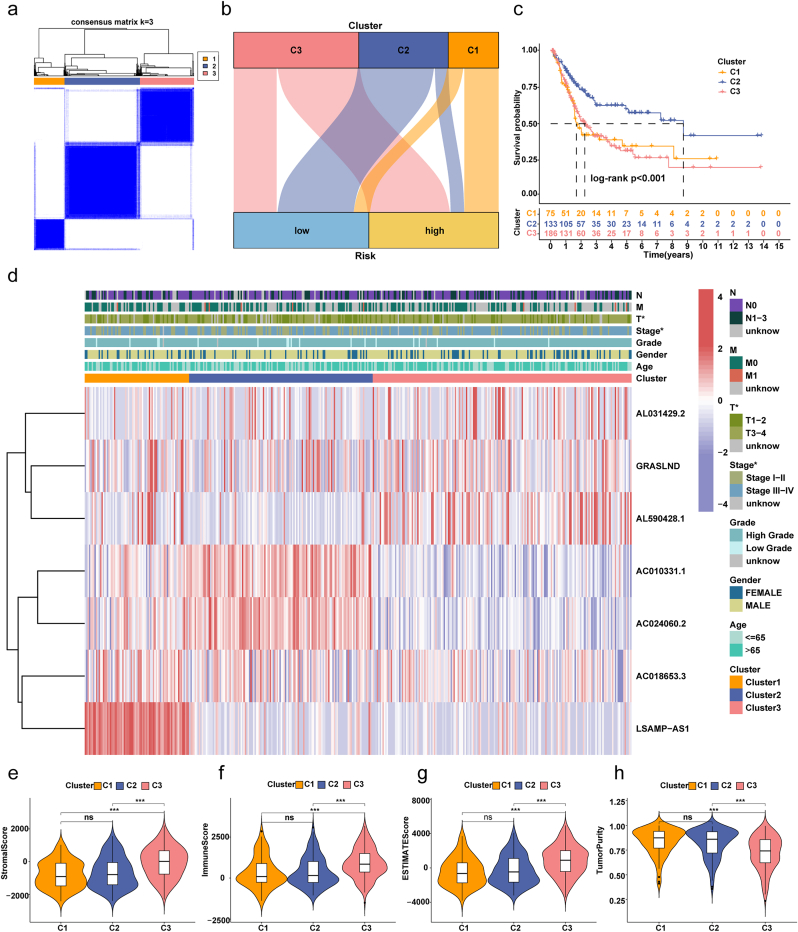
Dr-lncRNAs molecular patterns with OS and TME features. **(a)** Consensus score matrix from unsupervised clustering of 7 dr-lncRNAs expression profiles. **(b)** Sankey diagram showing cluster characteristics. **(c)** Kaplan-Meier curves of OS across the 3 clusters. **(d)** Heatmap of clinical characteristics and dr-lncRNA expression for the 3 clusters. **(e**–**h)** Differences in immune score, stromal score, estimate score, and tumor purity among the clusters. ∗P < 0.05, ∗∗P < 0.01, ∗∗∗P < 0.001.

**Fig. 7:**
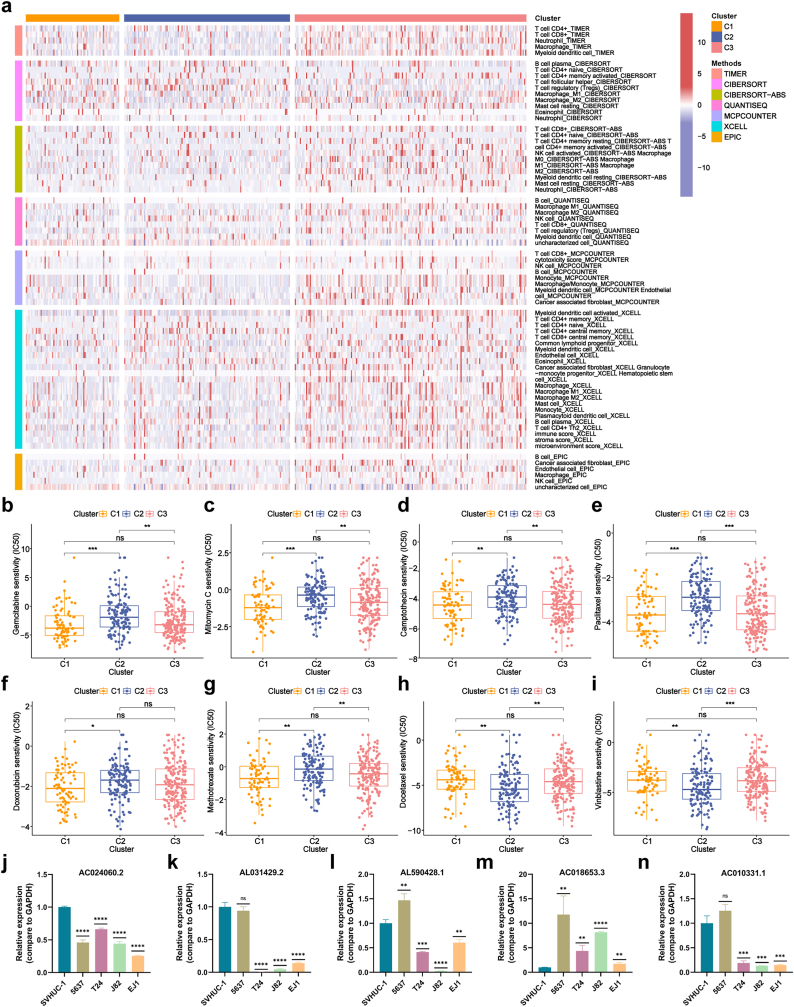
Integration of immune characteristics and validation of lncRNA expression by qRT-PCR. **(a)** Heatmap of immune cell expression across 3 clusters using 7 algorithms. **(b**–**i)** IC50 values for gemcitabine, mitomycin C, camptothecin, paclitaxel, doxorubicin, methotrexate, docetaxel, and vinblastine. **(j**–**n)** qRT-PCR validation of 7 dr-lncRNAs in four BLCA cell lines. ∗P < 0.05, ∗∗P < 0.01, ∗∗∗P < 0.001, ∗∗∗∗P < 0.0001.

**Supplementary Fig. S4:**
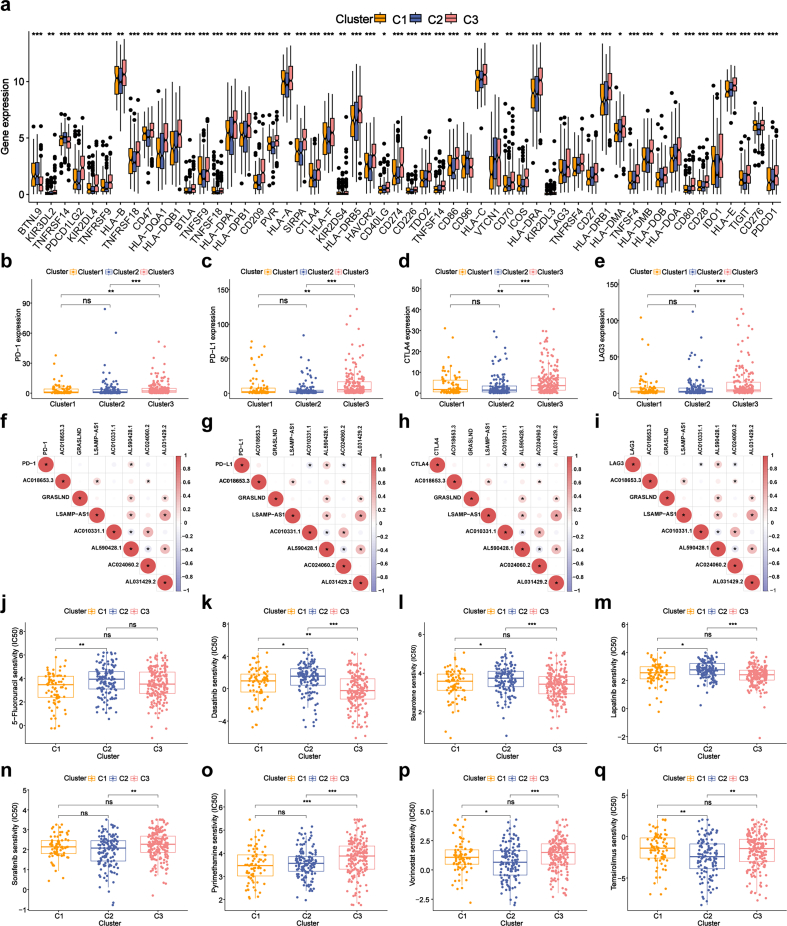
Immune Checkpoints and Drug Sensitivity Analysis. (a) Boxplots of immune checkpoint expression across 3 clusters. (b-e) Expression levels of immune checkpoint genes (PD-1, PD-L1, CTLA-4, LAG3) in 3 clusters. (f-i) Correlation between immune checkpoint genes (PD-1, PD-L1, CTLA-4, LAG3) and 7 survival-associated dr-lncRNAs. (j-q) IC50 values for 5-fluorouracil, dasatinib, bexarotene, lapatinib, sorafenib, pyrimethamine, vorinostat, and temsirolimus. ∗P < 0.05, ∗∗P < 0.01, ∗∗∗P < 0.001

To investigate the expression of 7 prognostic lncRNAs in BLCA, 394 patients were classified into 3 clusters using the “Consensus Cluster Plus” package. High-risk patients were predominantly in clusters 1 and 3, while low-risk patients were mainly in cluster 2 (Fig. 6a–b). Subsequently, a survival analysis was conducted to discern potential disparities in patient prognosis among these distinct clusters. Survival analysis revealed that Cluster 2 had the highest OS, while Clusters 1 and 3 showed lower OS (Fig. 6c). A heatmap of clinical characteristics and lncRNA expression highlighted that Cluster 2, associated with better prognosis, showed higher expression of AC010331.1, AC024060.2, and AC018653.3, which were inversely correlated with risk. In contrast, Clusters 1 and 3 exhibited higher levels of LSAMP-AS1, AL031429.2, GRASLND, and AL590428.1, correlating positively with risk (Fig. 6d). Further analysis of tumor microenvironment characteristics revealed that Cluster 3, despite poor prognosis, displayed the highest levels of immune cell infiltration and elevated expression of immune checkpoint genes (Fig. 6, Fig. 7a, Supplementary Fig. 4a–e). This immune-enriched but dysfunctional profile suggests an immune-exhausted state, which may contribute to tumor progression and immunotherapy resistance. In particular, AL590428.1 was strongly correlated with the expression of multiple immune checkpoint genes, indicating its potential role in modulating immune evasion and serving as a predictive biomarker for immunotherapy (Supplementary Fig. 4f–i). Moreover, the drug sensitivity analysis showed that clusters 1 and 3 had lower IC50 values for drugs like Gemcitabine, Mitomycin C, Camptothecin, Paclitaxel, Doxorubicin, and Methotrexate, suggesting better drug effectiveness in high-risk patients (Fig. 7b–g, Supplementary Fig. 4j–m). Conversely, cluster 2 had higher IC50 values for Docetaxel, Vinblastine, Sorafenib, Pyrimethamine, Vorinostat, and Temsirolimus (Fig. 7h–i, Supplementary Fig. 4n–q). These findings highlight the clinical relevance of molecular subtyping based on dr-lncRNAs, which may guide treatment selection and improve outcomes through tailored therapeutic strategies.

### 3.12 Validation of lncRNA expression profiles in BLCA cells

We used qRT-PCR to assess the expression of 7 selected lncRNAs in bladder cell lines SVHUC-1 and BLCA cell lines. The results revealed significant expression changes, with AC018653.3 showing upregulation in BLCA cells compared to SVHUC-1, while AC024060.2, AL031429.2, AL590428.1, and AC010331.1 were downregulated. Notably, AL590428.1 was upregulated only in the 5637 cell line, and AC010331.1 showed no significant expression change in 5637 cells. GRASLND and LSAMP-AS1 were undetectable due to low expression. Bioinformatics analysis indicated upregulation of AC024060.2 and AC010331.1 in BLCA samples, although some discrepancies with qRT-PCR results were observed (Fig. 7j–n). These findings highlight the complexity and potential molecular heterogeneity of lncRNA expression in BLCA, warranting further exploration of their roles and clinical relevance.

## 4 Discussion

BLCA incidence has increased globally, with a lifetime risk of 1.1 % for males and 0.27 % for females. Its pathogenesis is influenced by diverse etiological factors, including tobacco use, exposure to environmental carcinogens, occupational hazards, schistosomiasis infection, and genetic predispositions. These factors may exert their oncogenic influence by directly or indirectly altering lncRNA expression and function. For instance, Tobacco-derived carcinogens induce genomic instability and epigenetic alterations, potentially activating oncogenic lncRNAs or silencing tumour-suppressive lncRNAs [18,19]. Similarly, Schistosomiasis-induced chronic inflammation may also facilitate lncRNA-driven oncogenic signaling [20]. Furthermore, genetic predisposition has been shown to alter lncRNA expression patterns associated with disease progression and patient prognosis [21,22]. Increasing evidence highlights the role of lncRNAs in the initiation, progression, and metastasis of BLCA.

Disulfidoptosis is a novel and emerging form of regulated cell death characterized by abnormal disulfide accumulation and cytoskeletal protein crosslinking under glucose-deprived conditions. Despite its recognized significance in cancer cell viability and redox vulnerability, the role of lncRNAs regulating this process in BLCA remains unclear. In this study, we proposed and validated a novel risk model based on dr-lncRNAs that predicts prognosis and provides therapeutic insights for patients with BLCA. Through integrative analysis, 27 genes involved in disulfidoptosis were identified, from which 695 co-expressed lncRNAs were screened, and 403 were found to be differentially expressed in BLCA. Subsequent Cox and LASSO regression analyses led to the identification of 9 prognostic dr-lncRNAs, of which 7 were incorporated into the final model. Notably, GRASLND and AC010331.1 have previously been reported as prognostic markers in gastric and bladder cancers, respectively, consistent with our findings [23,24]. The remaining five lncRNAs, including LSAMP-AS1, AL59428.1, AL031429.2, AC08653.3, and AC024060.2, are reported here for the first time in association with BLCA prognosis, warranting further functional characterization.

The proposed model exhibited robust predictive performance across the entire cohort, training set, and test set. Patients in the high-risk group had significantly worse OS, and multivariate Cox regression confirmed the risk score as an independent prognostic factor, surpassing traditional clinical indicators such as age, gender, grade, and stage. ROC and calibration curves, along with a C-index of 0.712, further underscored the model's discriminatory power and accuracy. PCA demonstrated clear separation between high- and low-risk groups based on lncRNA expression, reinforcing the biological relevance of the identified signature.

To gain insight into the functional pathways associated with the prognostic lncRNAs, GO and KEGG enrichment analyses were performed on genes differentially expressed between the two risk groups. These genes were significantly enriched in immune-related biological processes, including humoral immune responses, leukocyte migration, and granulocyte chemotaxis, as well as pathways such as the IL-17 signaling and PI3K-AKT signaling cascades. Notably, these pathways have previously been implicated in BLCA progression and metastasis [[25], [26], [27], [28]].

Our findings revealed substantial disparities within the tumor immune microenvironment when comparing high-risk and low-risk groups. Higher expression levels of immune checkpoint genes were consistently observed in the high-risk group, including PD-1, PD-L1, CTLA-4, and LAG-3, which displayed a positive correlation with risk scores. CTLA-4, expressed in T cells and interacting with CD80/CD86, restricts T cell activation, leading to T cell exhaustion. Research has suggested that disrupting the expression of CTLA-4 in peripheral blood CD8^+^ T cells can enhance the cytotoxic immune response against bladder cancer cells, exerting anti-tumor effects [29]. The infiltration of LAG-3 has been associated with poor prognosis in muscle-invasive bladder cancer. In a study by Attalla et al., the upregulation of TIM-3 and TIGIT was linked to the suppression of NK cell effector function in patients with urothelial bladder carcinoma, and the overexpression of TIM-3 and TIGIT in peripheral blood NK cells may serve as biomarkers for assessing disease severity and monitoring treatment effectiveness [30]. Currently, numerous clinical trials involving immune checkpoint inhibitors for bladder cancer are underway, showing considerable promise in improving and maintaining the quality of life for bladder cancer patients, particularly those with severe comorbidities who are unable to undergo standard treatments [31,32].

TMB serves as a biomarker for predicting the immune response to immune checkpoint inhibitor therapy, with a positive correlation observed between elevated TMB levels and improved survival outcomes. In this context, TMB analysis revealed a positive correlation between higher TMB levels and improved responses to immune checkpoint blockade. TP53 mutations, which are common in BLCA, were also associated with enhanced responses to immunotherapy. Genetic mutations, such as those in RB1 and TP53, were associated with increased immune cell infiltration, particularly CD8^+^ T cells, suggesting that immune modulation could be a promising treatment strategy.

For individuals with BLCA who are not suitable candidates for surgery, traditional radiotherapy remains a viable treatment option. However, recent trials have shown promising results for the use of concurrent chemoradiotherapy or immunotherapy as alternative treatment approaches [[33], [34], [35], [36]]. Drug sensitivity analysis has revealed that patients in the high-risk group exhibit heightened sensitivity to gemcitabine, ixabepilone, paclitaxel, docetaxel, methotrexate, and 5-fluorouracil. Gemcitabine, a cytidine nucleoside analog, when administered intravesically postoperatively, can effectively reduce the risk of recurrence in non-muscle-invasive bladder cancer [37]. Paclitaxel, a natural anti-tumor agent widely employed in chemotherapy for various cancers, may also modulate multiple immune cells, thereby contributing to tumor immunotherapy [38]. Both docetaxel and methotrexate, known as anti-metabolite drugs, disrupt DNA and RNA synthesis. A novel adjuvant chemotherapy regimen, dose-dense Methotrexate, Vinblastine, Doxorubicin, and Cisplatin, is believed to offer improved tumor control and extended survival in muscle-invasive bladder cancer [39,40]. In summary, the research outcomes provide robust scientific evidence to guide the selection of chemotherapy agents in clinical practice.

The process of unsupervised consensus clustering is utilized to categorize individuals with BLCA into three distinct subtypes, with Cluster 3 being particularly noteworthy due to its association with the lowest survival prognosis. The observed cluster exhibits notable differences in lncRNAs expression, specifically showing elevated levels of AL031429.2, GRASLND, and AL590428.1. Despite the worst prognosis, Cluster 3 demonstrates heightened levels of immune cell infiltration, immunological functioning, and immune checkpoint gene expression, indicating the potential advantages of employing immunotherapeutic approaches. The existence of diverse drug sensitivities among different subtypes of BLCA provides a foundation for the development of personalized therapeutic approaches.

Notably, qRT-PCR validation revealed partial concordance with the bioinformatics results. For instance, AL031429.2 and AL590428.1 were downregulated in most cell lines, whereas AC018653.3 was upregulated. GRASLND and LSAMP-AS1 were undetectable due to low expression levels. These discrepancies may be attributed to tumor heterogeneity, cell line-specific expression patterns, and differences between in vitro and in vivo contexts. Moreover, inconsistencies between transcriptomic data and qRT-PCR results are not uncommon and could reflect technical variability, sample-type differences, or post-transcriptional regulation mechanisms. These findings highlight the need for additional validation in larger cohorts and primary tumor samples to reconcile computational predictions with experimental observations, and to fully elucidate the clinical relevance of dr-lncRNAs in BLCA in future studies.

Despite the promising findings, several limitations should be acknowledged. First, the study is based solely on data derived from the TCGA cohort, which may introduce dataset-specific biases and limit the broader applicability of the findings. Second, external validation using independent patient cohorts was not performed, potentially affecting the generalizability of the risk model. Third, although qRT-PCR validation was conducted for selected lncRNAs, in-depth mechanistic studies are still required to elucidate their biological functions in BLCA. Future research should prioritize experimental validation and include larger, more diverse clinical samples to enhance the robustness and dependability of these findings.

In summary, our study presents a robust dr-lncRNA-based risk model that not only predicts patient prognosis but also informs immunological and therapeutic decision-making in BLCA. By integrating transcriptomic and clinical data, the model offers a valuable tool for risk stratification and personalized treatment in bladder cancer management.

## 5 Conclusion

This study identifies a novel dr-lncRNAs signature in BLCA, providing valuable insights for risk stratification, immune analysis, and personalized treatment strategies, offering robust data support for clinical decision-making in personalized therapy.

## CRediT authorship contribution statement

**Zhixiong Zhang:** Writing – review & editing, Writing – original draft, Validation, Formal analysis, Conceptualization. **Jinghua Zhong:** Writing – review & editing, Writing – original draft, Visualization, Data curation. **Muhammad Sarfaraz Iqbal:** Writing – review & editing, Writing – original draft, Visualization, Formal analysis. **Zhiwen Zeng:** Writing – review & editing, Project administration, Conceptualization. **Xiaolu Duan:** Writing – review & editing, Funding acquisition, Conceptualization.

## Data availability

The datasets analyzed in this study are available in The Cancer Genome Atlas (TCGA, https://portal.gdc.cancer.gov/) database and in the Supplementary Materials.

### Funding

This work was funded by grants from the National Natural Science Foundation of China (No. 81872437, No. 81402430), the Characteristic Innovation project of Guangdong Province Education Department (2019KTSCX140), and the Scientific research projects in colleges and universities of Guangzhou Education Bureau (No. 201831811).

## Declaration of competing interest

All authors disclosed no relevant relationships.
