# Supplementary material for: A comprehensive risk model of disulfidoptosis-related lncRNAs predicts prognosis and therapeutic implications in bladder cancer
Source: Biochem Biophys Rep. 2025 May 26;42:102060. doi: 10.1016/j.bbrep.2025.102060 (PMC12159218; doi:10.1016/j.bbrep.2025.102060)
Supplement: Multimedia component 1 [file mmc1.docx]

**Supplementary Table S1**

| **ID** |
| --- |
| OXSM |
| SLC7A11 |
| GYS1 |
| NDUFS1 |
| NDUFA11 |
| NUBPL |
| NCKAP1 |
| LRPPRC |
| SLC3A2 |
| RPN1 |
| ACTN4 |
| ACTB |
| CD2AP |
| CAPZB |
| DSTN |
| FLNA |
| FLNB |
| INF2 |
| IQGAP1 |
| MYH10 |
| MYL6 |
| MYH9 |
| PDLIM1 |
| TLN1 |
| PRDX1 |
| ACTN1 |
| TLN2 |

27 disulfidptosis-related genes.
