# Supplementary material for: A comprehensive risk model of disulfidoptosis-related lncRNAs predicts prognosis and therapeutic implications in bladder cancer
Source: Biochem Biophys Rep. 2025 May 26;42:102060. doi: 10.1016/j.bbrep.2025.102060 (PMC12159218; doi:10.1016/j.bbrep.2025.102060)
Supplement: Multimedia component 2 [file mmc2.docx]

**Supplementary Table S2**

| **ID** | **Sequences** |
| --- | --- |
| AC018653.3 | Forward: 5'- ATCCTGGCATTTGTAGCCCA-3'  Reverse: 5'-TCTCTCGAACCTGCCATCGT-3' |
| GRASLND | Forward: 5'- ACCACGAACTTTGGAGTGGA-3'  Reverse: 5'-TGTGTTCCCTCGGTGTAGGA-3' |
| LSAMP-AS1 | Forward: 5'- CAGAGGGATGCCCACAGAGT-3'  Reverse: 5'-CTGGTGGCTCTTCTCATCCTTT-3' |
| AC010331.1 | Forward: 5'- GTGGCTCGATCACTCACCC-3'  Reverse: 5'- GAAACGGACCCGACACCAC-3' |
| AL590428.1 | Forward: 5'- CAGTTAGGGTTGGGTTGGCT-3'  Reverse: 5'-TTTCTGGCAGTTCCCTGCTT-3' |
| AC024060.2 | Forward: 5'- AGCAGTTTCCTTACCTTCTTGA-3'  Reverse: 5'-AGGGCCAAAGTAGGACATGC-3' |
| AL031429.2 | Forward: 5'- CTTGTGCCTGAGTTTTGGTG-3'  Reverse: 5'-TTTGACAGAGCTTCTCCCTTCA-3' |
| GAPDH | Forward: 5'- GGACCTGACCTGCCGTCTAG-3'  Reverse: 5'-GTAGCCCAGGATGCCCTTGA-3' |

The primers sequences for 7 disulfidptosis-related lncRNAs.
