# Supplementary material for: A comprehensive risk model of disulfidoptosis-related lncRNAs predicts prognosis and therapeutic implications in bladder cancer
Source: Biochem Biophys Rep. 2025 May 26;42:102060. doi: 10.1016/j.bbrep.2025.102060 (PMC12159218; doi:10.1016/j.bbrep.2025.102060)
Supplement: Multimedia component 3 [file mmc3.docx]

**Supplementary Table S3**

| Disulfidptosis Genes | lncRNA | Correlation | p-value | Regulation |
| --- | --- | --- | --- | --- |
| CD2AP | AL162727.2 | 0.44724221 | 1.18E-21 | postive |
| SLC7A11 | AC009268.2 | 0.403246137 | 1.53E-17 | postive |
| CD2AP | AC026771.1 | 0.405139605 | 1.05E-17 | postive |
| SLC7A11 | SAP30L-AS1 | 0.498764002 | 2.68E-27 | postive |
| CD2AP | AC104564.3 | 0.405030272 | 1.07E-17 | postive |
| CD2AP | AC069547.2 | 0.429741171 | 6.03E-20 | postive |
| NUBPL | AC120114.1 | 0.442979564 | 3.14E-21 | postive |
| CD2AP | GARS1-DT | 0.421753922 | 3.37E-19 | postive |
| SLC7A11 | AC021205.3 | 0.693592494 | 2.24E-60 | postive |
| CD2AP | AC006001.2 | 0.412939424 | 2.14E-18 | postive |
| SLC7A11 | AC107308.1 | 0.447892502 | 1.01E-21 | postive |
| NDUFA11 | AC098484.2 | 0.406297104 | 8.29E-18 | postive |
| SLC7A11 | NCK1-DT | 0.527821906 | 6.26E-31 | postive |
| CD2AP | AC008114.1 | 0.410402645 | 3.60E-18 | postive |
| MYH10 | MAP3K4-AS1 | 0.482479555 | 2.07E-25 | postive |
| CD2AP | C21orf62-AS1 | 0.514072886 | 3.62E-29 | postive |
| SLC7A11 | OSMR-AS1 | 0.517068491 | 1.52E-29 | postive |
| CD2AP | PDXP-DT | 0.486634372 | 6.98E-26 | postive |
| DSTN | AL513217.1 | 0.546054513 | 2.15E-33 | postive |
| FLNA | AL513217.1 | 0.591254614 | 3.36E-40 | postive |
| ACTB | ZNF436-AS1 | -0.432344593 | 3.40E-20 | negative |
| CD2AP | AC012459.1 | 0.465329062 | 1.58E-23 | postive |
| TLN2 | PTPRD-AS1 | 0.505427384 | 4.23E-28 | postive |
| SLC7A11 | DST-AS1 | 0.50420249 | 5.95E-28 | postive |
| SLC7A11 | AC104472.5 | 0.466959274 | 1.06E-23 | postive |
| CD2AP | AC114956.2 | 0.494122787 | 9.48E-27 | postive |
| ACTB | AC006042.1 | -0.43349356 | 2.64E-20 | negative |
| OXSM | AL139287.1 | 0.4025972 | 1.74E-17 | postive |
| CD2AP | RNASEH2B-AS1 | 0.410789425 | 3.33E-18 | postive |
| CD2AP | AC018616.1 | 0.43865927 | 8.35E-21 | postive |
| ACTB | AL133410.1 | -0.401377733 | 2.22E-17 | negative |
| NDUFA11 | GATA3-AS1 | 0.507927687 | 2.09E-28 | postive |
| ACTB | GATA3-AS1 | -0.403278395 | 1.52E-17 | negative |
| CD2AP | AC006566.1 | 0.464994781 | 1.71E-23 | postive |
| ACTB | SOCAR | 0.432548764 | 3.26E-20 | postive |
| CD2AP | AC024267.3 | 0.492831781 | 1.34E-26 | postive |
| NDUFA11 | AC010618.2 | 0.55563525 | 9.46E-35 | postive |
| MYH10 | LINC01775 | 0.469527626 | 5.59E-24 | postive |
| TLN1 | LINC01775 | 0.489844244 | 2.98E-26 | postive |
| FLNA | AC131097.3 | 0.526390841 | 9.64E-31 | postive |
| TLN1 | AC131097.3 | 0.635434786 | 5.62E-48 | postive |
| MYH10 | AC007541.1 | 0.456434497 | 1.36E-22 | postive |
| SLC7A11 | AL138999.1 | 0.713919686 | 1.94E-65 | postive |
| SLC7A11 | C1QTNF7-AS1 | 0.711458853 | 8.41E-65 | postive |
| SLC7A11 | AP003900.1 | 0.736541635 | 1.28E-71 | postive |
| LRPPRC | SNHG29 | 0.415883038 | 1.16E-18 | postive |
| CD2AP | AC078778.1 | 0.405947369 | 8.90E-18 | postive |
| NUBPL | AL035411.3 | 0.412326965 | 2.42E-18 | postive |
| NCKAP1 | AL035411.3 | 0.450193376 | 5.93E-22 | postive |
| CD2AP | AL035411.3 | 0.576493422 | 7.34E-38 | postive |
| SLC7A11 | AC079921.2 | 0.620266246 | 3.63E-45 | postive |
| TLN1 | AC079921.2 | 0.477230098 | 8.01E-25 | postive |
| NDUFA11 | AC103706.1 | 0.450835518 | 5.10E-22 | postive |
| CD2AP | AC004882.3 | 0.51493223 | 2.82E-29 | postive |
| SLC7A11 | AC099811.5 | 0.442655632 | 3.38E-21 | postive |
| SLC7A11 | AC074194.1 | 0.673095925 | 1.11E-55 | postive |
| ACTB | LINC01943 | 0.43486066 | 1.95E-20 | postive |
| ACTB | C10orf55 | 0.427743928 | 9.31E-20 | postive |
| ACTN1 | C10orf55 | 0.427328411 | 1.02E-19 | postive |
| NDUFA11 | CAHM | 0.402995789 | 1.61E-17 | postive |
| SLC7A11 | AC009269.4 | 0.439725882 | 6.57E-21 | postive |
| TLN1 | MACORIS | 0.491380256 | 1.98E-26 | postive |
| RPN1 | AL441992.1 | 0.443512605 | 2.78E-21 | postive |
| SLC7A11 | KLF7-IT1 | 0.561452888 | 1.35E-35 | postive |
| SLC7A11 | AC008750.4 | 0.49855235 | 2.84E-27 | postive |
| CD2AP | PABPC4-AS1 | 0.432195358 | 3.52E-20 | postive |
| FLNA | AC007671.1 | 0.518160474 | 1.10E-29 | postive |
| FLNA | AC120498.10 | 0.468973544 | 6.41E-24 | postive |
| CD2AP | AC009754.1 | 0.410115659 | 3.82E-18 | postive |
| TLN1 | TRG-AS1 | 0.45594533 | 1.53E-22 | postive |
| SLC7A11 | AC080188.1 | 0.602075085 | 5.41E-42 | postive |
| SLC7A11 | RNF216-IT1 | 0.477355387 | 7.76E-25 | postive |
| CD2AP | SPAG5-AS1 | 0.492769723 | 1.36E-26 | postive |
| SLC7A11 | NEXN-AS1 | 0.508261391 | 1.90E-28 | postive |
| TLN1 | NEXN-AS1 | 0.427821348 | 9.15E-20 | postive |
| CD2AP | AC007622.2 | 0.471932138 | 3.06E-24 | postive |
| MYH10 | AC108075.1 | 0.716640383 | 3.78E-66 | postive |
| NDUFA11 | AC120053.1 | 0.435429198 | 1.72E-20 | postive |
| SLC7A11 | AC104984.2 | 0.425194244 | 1.61E-19 | postive |
| NCKAP1 | AC018653.3 | 0.401960392 | 1.98E-17 | postive |
| NDUFA11 | AL355353.1 | 0.523069109 | 2.60E-30 | postive |
| SLC7A11 | FAM155A-IT1 | 0.723792145 | 4.65E-68 | postive |
| TLN2 | LINC01558 | 0.401469342 | 2.18E-17 | postive |
| SLC7A11 | AC087286.2 | 0.470871548 | 4.00E-24 | postive |
| NDUFA11 | RPARP-AS1 | 0.515457709 | 2.42E-29 | postive |
| CD2AP | AL121652.1 | 0.412909462 | 2.15E-18 | postive |
| TLN1 | BNC2-AS1 | 0.41937951 | 5.57E-19 | postive |
| CD2AP | LINC00412 | 0.404104878 | 1.29E-17 | postive |
| SLC7A11 | AL133445.2 | 0.602609364 | 4.39E-42 | postive |
| TLN2 | ID2-AS1 | 0.514979467 | 2.79E-29 | postive |
| CD2AP | AC010422.4 | 0.400176583 | 2.82E-17 | postive |
| NDUFA11 | AC067852.2 | 0.523367459 | 2.38E-30 | postive |
| CD2AP | AC007684.2 | 0.413849409 | 1.77E-18 | postive |
| CD2AP | AC010186.3 | 0.414176855 | 1.65E-18 | postive |
| CD2AP | AC245884.10 | 0.408806749 | 4.99E-18 | postive |
| MYH10 | AL121821.2 | 0.420222468 | 4.66E-19 | postive |
| CD2AP | WASHC5-AS1 | 0.445235719 | 1.87E-21 | postive |
| SLC7A11 | AL353804.2 | 0.453203032 | 2.93E-22 | postive |
| NDUFA11 | LINC01089 | 0.542164242 | 7.43E-33 | postive |
| SLC7A11 | Z99289.1 | 0.48792118 | 4.97E-26 | postive |
| SLC7A11 | AC012568.1 | 0.450119462 | 6.04E-22 | postive |
| SLC7A11 | AP000829.1 | 0.616909943 | 1.45E-44 | postive |
| SLC7A11 | LINC00513 | 0.551565795 | 3.61E-34 | postive |
| CD2AP | AC073957.3 | 0.498320138 | 3.03E-27 | postive |
| CD2AP | AL445309.1 | 0.431996163 | 3.68E-20 | postive |
| CD2AP | AC093535.1 | 0.480032717 | 3.90E-25 | postive |
| CD2AP | PSPC1-AS2 | 0.460631682 | 4.96E-23 | postive |
| SLC7A11 | AL137186.1 | 0.417897326 | 7.61E-19 | postive |
| CD2AP | MCM3AP-AS1 | 0.411908051 | 2.64E-18 | postive |
| SLC7A11 | PART1 | 0.525715215 | 1.18E-30 | postive |
| SLC7A11 | LINC02256 | 0.46456999 | 1.90E-23 | postive |
| IQGAP1 | LINC02256 | 0.406837016 | 7.44E-18 | postive |
| TLN1 | LINC02256 | 0.451066703 | 4.84E-22 | postive |
| DSTN | MIR100HG | 0.45355795 | 2.69E-22 | postive |
| FLNA | MIR100HG | 0.654523925 | 9.59E-52 | postive |
| TLN1 | MIR100HG | 0.563748291 | 6.20E-36 | postive |
| ACTN1 | MIR100HG | 0.524904381 | 1.50E-30 | postive |
| CD2AP | AC092828.1 | 0.437974592 | 9.74E-21 | postive |
| TLN2 | SATB2-AS1 | 0.415306719 | 1.31E-18 | postive |
| CD2AP | AC022165.1 | 0.422001641 | 3.20E-19 | postive |
| CD2AP | AC078795.1 | 0.471171993 | 3.71E-24 | postive |
| SLC7A11 | TCF4-AS1 | 0.695193228 | 9.27E-61 | postive |
| PRDX1 | TCF4-AS1 | 0.410386416 | 3.61E-18 | postive |
| NDUFA11 | AC009065.8 | 0.448783736 | 8.24E-22 | postive |
| NDUFS1 | AC005670.3 | 0.505858377 | 3.75E-28 | postive |
| NUBPL | AC005670.3 | 0.516257017 | 1.92E-29 | postive |
| NCKAP1 | AC005670.3 | 0.421197175 | 3.79E-19 | postive |
| TLN2 | AC005670.3 | 0.631706472 | 2.85E-47 | postive |
| NDUFA11 | AC008915.2 | 0.458331857 | 8.63E-23 | postive |
| LRPPRC | EMSLR | 0.446033243 | 1.56E-21 | postive |
| FLNA | AC000403.1 | 0.404741694 | 1.13E-17 | postive |
| CD2AP | WARS2-AS1 | 0.417023448 | 9.14E-19 | postive |
| CD2AP | AC012358.1 | 0.406357879 | 8.19E-18 | postive |
| CD2AP | ZNF32-AS2 | 0.470759853 | 4.11E-24 | postive |
| SLC7A11 | AL512656.1 | 0.428576271 | 7.77E-20 | postive |
| NDUFA11 | SNHG12 | 0.468617745 | 7.01E-24 | postive |
| SLC7A11 | AL138759.1 | 0.602294566 | 4.97E-42 | postive |
| CD2AP | AC003070.1 | 0.400600717 | 2.59E-17 | postive |
| SLC7A11 | AL353593.1 | 0.549908647 | 6.19E-34 | postive |
| TLN2 | HNF1A-AS1 | 0.542881378 | 5.92E-33 | postive |
| CD2AP | AC073130.2 | 0.412640408 | 2.27E-18 | postive |
| SLC7A11 | AC073569.3 | 0.437978307 | 9.73E-21 | postive |
| SLC7A11 | AL031651.2 | 0.640519208 | 5.91E-49 | postive |
| TLN1 | AL031651.2 | 0.414800086 | 1.45E-18 | postive |
| MYH10 | GRASLND | 0.433217396 | 2.81E-20 | postive |
| CD2AP | AC007128.1 | 0.438682864 | 8.31E-21 | postive |
| SLC7A11 | GRK5-IT1 | 0.614603092 | 3.71E-44 | postive |
| CD2AP | AL157838.1 | 0.48151413 | 2.66E-25 | postive |
| SLC7A11 | AC104564.5 | 0.427991288 | 8.82E-20 | postive |
| SLC7A11 | LRIG2-DT | 0.651194473 | 4.55E-51 | postive |
| NDUFA11 | AL121832.2 | 0.429113999 | 6.91E-20 | postive |
| TLN2 | AC104534.1 | 0.622367449 | 1.51E-45 | postive |
| NDUFA11 | AC027682.4 | 0.432077083 | 3.61E-20 | postive |
| SLC7A11 | AC092807.3 | 0.532237484 | 1.64E-31 | postive |
| SLC7A11 | FAM230C | 0.474974473 | 1.42E-24 | postive |
| CD2AP | AL031666.2 | 0.423462216 | 2.34E-19 | postive |
| CD2AP | LINC00161 | 0.406521496 | 7.93E-18 | postive |
| SLC7A11 | STARD13-IT1 | 0.646594265 | 3.80E-50 | postive |
| FLNA | AL132642.1 | 0.451114703 | 4.78E-22 | postive |
| NDUFA11 | TP53TG1 | 0.477387391 | 7.69E-25 | postive |
| ACTB | CYTOR | 0.428566715 | 7.78E-20 | postive |
| CD2AP | AL359878.1 | 0.484408217 | 1.25E-25 | postive |
| FLNA | AL109741.1 | 0.422531147 | 2.86E-19 | postive |
| TLN1 | AL109741.1 | 0.474599938 | 1.56E-24 | postive |
| NDUFA11 | AC005387.1 | 0.603144074 | 3.57E-42 | postive |
| CD2AP | FMR1-IT1 | 0.479995313 | 3.94E-25 | postive |
| SLC7A11 | AC080023.1 | 0.465506106 | 1.51E-23 | postive |
| SLC7A11 | AC118555.1 | 0.653746667 | 1.38E-51 | postive |
| FLNA | AC103591.4 | 0.449222505 | 7.44E-22 | postive |
| TLN1 | AC103591.4 | 0.513426728 | 4.36E-29 | postive |
| DSTN | PGM5-AS1 | 0.416489061 | 1.02E-18 | postive |
| FLNA | PGM5-AS1 | 0.448357032 | 9.10E-22 | postive |
| DSTN | AP001107.5 | 0.402781814 | 1.68E-17 | postive |
| FLNA | AP001107.5 | 0.485681104 | 8.97E-26 | postive |
| FLNA | AC120498.2 | 0.410298795 | 3.68E-18 | postive |
| SLC7A11 | AC090673.1 | 0.620905795 | 2.78E-45 | postive |
| SLC7A11 | AC025031.2 | 0.454170817 | 2.33E-22 | postive |
| DSTN | LINC00702 | 0.581656571 | 1.15E-38 | postive |
| FLNA | LINC00702 | 0.699236544 | 9.72E-62 | postive |
| TLN1 | LINC00702 | 0.492689905 | 1.39E-26 | postive |
| ACTN1 | LINC00702 | 0.607095671 | 7.54E-43 | postive |
| CD2AP | AL117329.1 | 0.414226025 | 1.64E-18 | postive |
| CD2AP | MIR29B2CHG | 0.495215389 | 7.05E-27 | postive |
| SLC7A11 | SALRNA1 | 0.579518564 | 2.49E-38 | postive |
| CD2AP | LINC00894 | 0.438009632 | 9.66E-21 | postive |
| SLC7A11 | AC130371.1 | 0.656418757 | 3.91E-52 | postive |
| CD2AP | AL078459.1 | 0.445135999 | 1.91E-21 | postive |
| CD2AP | AC079160.1 | 0.450059813 | 6.12E-22 | postive |
| CD2AP | AC010615.2 | 0.490288479 | 2.65E-26 | postive |
| CD2AP | AP001625.2 | 0.494593107 | 8.35E-27 | postive |
| CD2AP | AC110611.1 | 0.488131044 | 4.70E-26 | postive |
| SLC7A11 | AL353804.1 | 0.493293687 | 1.19E-26 | postive |
| CD2AP | AL031666.3 | 0.450800564 | 5.15E-22 | postive |
| CD2AP | CDC42-IT1 | 0.426029426 | 1.35E-19 | postive |
| CD2AP | C1orf220 | 0.476806856 | 8.92E-25 | postive |
| SLC7A11 | AC009716.1 | 0.611958783 | 1.08E-43 | postive |
| CD2AP | AL109923.1 | 0.402238427 | 1.87E-17 | postive |
| CD2AP | AC109460.2 | 0.425521774 | 1.50E-19 | postive |
| CD2AP | LNX1-AS2 | 0.402957322 | 1.62E-17 | postive |
| CD2AP | AL391840.3 | 0.400023695 | 2.90E-17 | postive |
| CD2AP | AP000873.2 | 0.430270786 | 5.37E-20 | postive |
| CD2AP | MAP3K5-AS1 | 0.402980204 | 1.61E-17 | postive |
| CD2AP | Z94721.3 | 0.45269573 | 3.30E-22 | postive |
| SLC7A11 | LINC02577 | 0.46930975 | 5.90E-24 | postive |
| SLC7A11 | LSAMP-AS1 | 0.657912183 | 1.92E-52 | postive |
| SLC7A11 | GSN-AS1 | 0.500222959 | 1.80E-27 | postive |
| ACTB | LNCTAM34A | -0.420900588 | 4.04E-19 | negative |
| SLC7A11 | AC025280.3 | 0.470290319 | 4.62E-24 | postive |
| ACTB | AL359220.1 | -0.452971152 | 3.09E-22 | negative |
| ACTB | GAS6-DT | 0.458187229 | 8.94E-23 | postive |
| FLNA | GAS6-DT | 0.544129083 | 3.98E-33 | postive |
| TLN1 | GAS6-DT | 0.49158935 | 1.87E-26 | postive |
| ACTN1 | GAS6-DT | 0.542932797 | 5.82E-33 | postive |
| NDUFA11 | AC008610.1 | 0.607011357 | 7.80E-43 | postive |
| CD2AP | AC079753.2 | 0.419231486 | 5.75E-19 | postive |
| SLC7A11 | RBM5-AS1 | 0.449170005 | 7.53E-22 | postive |
| SLC7A11 | LINC00536 | 0.650492078 | 6.31E-51 | postive |
| CD2AP | AC012645.2 | 0.409354518 | 4.46E-18 | postive |
| SLC7A11 | IQCJ-SCHIP1-AS1 | 0.639665552 | 8.66E-49 | postive |
| CD2AP | AC106845.1 | 0.432176916 | 3.53E-20 | postive |
| NUBPL | AL162171.1 | 0.400448046 | 2.67E-17 | postive |
| TLN1 | LINC00996 | 0.452156199 | 3.74E-22 | postive |
| CD2AP | AL121832.3 | 0.45308799 | 3.01E-22 | postive |
| CD2AP | AC010789.2 | 0.436962184 | 1.22E-20 | postive |
| NDUFA11 | AC145423.1 | 0.450507555 | 5.51E-22 | postive |
| CD2AP | AC008115.3 | 0.416848255 | 9.48E-19 | postive |
| CD2AP | LINC02042 | 0.427550344 | 9.71E-20 | postive |
| CD2AP | ZDHHC20-IT1 | 0.486339795 | 7.55E-26 | postive |
| CD2AP | AC023794.1 | 0.430597773 | 5.00E-20 | postive |
| CD2AP | AC018766.1 | 0.417610362 | 8.08E-19 | postive |
| SLC7A11 | AC012358.2 | 0.464568526 | 1.90E-23 | postive |
| SLC7A11 | AL356234.3 | 0.518359075 | 1.04E-29 | postive |
| CD2AP | AC060780.1 | 0.434496486 | 2.12E-20 | postive |
| SLC7A11 | AL139424.3 | 0.584282951 | 4.43E-39 | postive |
| NUBPL | AL031670.1 | 0.41021669 | 3.74E-18 | postive |
| SLC7A11 | Z98885.3 | 0.409366155 | 4.45E-18 | postive |
| CD2AP | AC040934.1 | 0.445247221 | 1.87E-21 | postive |
| FLNA | LINC01352 | 0.404965685 | 1.08E-17 | postive |
| TLN1 | LINC01352 | 0.402742178 | 1.69E-17 | postive |
| NCKAP1 | OGFRP1 | 0.422483013 | 2.89E-19 | postive |
| CD2AP | AC002128.1 | 0.484983227 | 1.08E-25 | postive |
| SLC7A11 | AC020978.2 | 0.50906018 | 1.52E-28 | postive |
| CD2AP | AC010201.2 | 0.438918571 | 7.88E-21 | postive |
| CD2AP | AC012557.1 | 0.408753237 | 5.04E-18 | postive |
| SLC7A11 | LINC02649 | 0.438772234 | 8.14E-21 | postive |
| CD2AP | AC011503.2 | 0.431748668 | 3.88E-20 | postive |
| NDUFA11 | ARHGAP27P1-BPTFP1-KPNA2P3 | 0.414044527 | 1.70E-18 | postive |
| SLC7A11 | AL359762.3 | 0.412004017 | 2.59E-18 | postive |
| SLC7A11 | AC025280.1 | 0.440163064 | 5.95E-21 | postive |
| CD2AP | LINC01675 | 0.406512415 | 7.94E-18 | postive |
| DSTN | AC053503.3 | 0.632205852 | 2.29E-47 | postive |
| FLNA | AC053503.3 | 0.710608589 | 1.39E-64 | postive |
| TLN1 | AC053503.3 | 0.452178855 | 3.72E-22 | postive |
| ACTN1 | AC053503.3 | 0.469021621 | 6.34E-24 | postive |
| SLC7A11 | AC104984.5 | 0.460765628 | 4.80E-23 | postive |
| CD2AP | AC005856.1 | 0.426233548 | 1.29E-19 | postive |
| CD2AP | AC007128.2 | 0.484077586 | 1.37E-25 | postive |
| SLC7A11 | AL136320.1 | 0.616329712 | 1.84E-44 | postive |
| CD2AP | AC005828.1 | 0.446672397 | 1.34E-21 | postive |
| SLC7A11 | LINC01687 | 0.457698567 | 1.00E-22 | postive |
| NDUFA11 | SNHG11 | 0.458235636 | 8.83E-23 | postive |
| SLC7A11 | AC063944.2 | 0.476880537 | 8.76E-25 | postive |
| NDUFA11 | AC087741.1 | 0.434016356 | 2.35E-20 | postive |
| ACTB | AC087741.1 | -0.422069463 | 3.15E-19 | negative |
| CD2AP | AP000766.1 | 0.53435321 | 8.53E-32 | postive |
| CD2AP | LIPC-AS1 | 0.420659603 | 4.25E-19 | postive |
| CD2AP | N4BP2L2-IT2 | 0.431396143 | 4.19E-20 | postive |
| CD2AP | HCG25 | 0.439006617 | 7.72E-21 | postive |
| CD2AP | AC009262.1 | 0.431620594 | 3.99E-20 | postive |
| SLC7A11 | Z69666.1 | 0.497059886 | 4.27E-27 | postive |
| CD2AP | AC008781.2 | 0.400674362 | 2.55E-17 | postive |
| NDUFA11 | AC090425.2 | 0.470363038 | 4.54E-24 | postive |
| SLC7A11 | AC067852.3 | 0.4577057 | 1.00E-22 | postive |
| CD2AP | AL021878.4 | 0.471299368 | 3.59E-24 | postive |
| CD2AP | AC021491.2 | 0.466278205 | 1.25E-23 | postive |
| CD2AP | AC005972.3 | 0.427571028 | 9.66E-20 | postive |
| CD2AP | AL512506.1 | 0.433748457 | 2.50E-20 | postive |
| SLC7A11 | AC004832.4 | 0.480133122 | 3.80E-25 | postive |
| NDUFA11 | AC112484.3 | 0.404093839 | 1.29E-17 | postive |
| NDUFA11 | SBNO1-AS1 | 0.400856187 | 2.46E-17 | postive |
| CD2AP | CCNT2-AS1 | 0.414312654 | 1.61E-18 | postive |
| PRDX1 | AL136018.1 | 0.466028198 | 1.33E-23 | postive |
| CD2AP | AL513327.2 | 0.412630463 | 2.28E-18 | postive |
| CD2AP | PCAT1 | 0.403683219 | 1.40E-17 | postive |
| SLC7A11 | AC058791.1 | 0.5086512 | 1.70E-28 | postive |
| NDUFS1 | AL356019.2 | 0.436945564 | 1.23E-20 | postive |
| NUBPL | AL356019.2 | 0.525444753 | 1.28E-30 | postive |
| NCKAP1 | AL356019.2 | 0.448274684 | 9.27E-22 | postive |
| CD2AP | NARF-IT1 | 0.408518407 | 5.29E-18 | postive |
| FLNA | AC093849.2 | 0.401151379 | 2.32E-17 | postive |
| CD2AP | AC092338.3 | 0.41102643 | 3.17E-18 | postive |
| CD2AP | AL353697.1 | 0.403366107 | 1.49E-17 | postive |
| CD2AP | AL442636.1 | 0.43762171 | 1.05E-20 | postive |
| SLC7A11 | AL031600.2 | 0.424507624 | 1.87E-19 | postive |
| CD2AP | AP001001.1 | 0.431817667 | 3.82E-20 | postive |
| DSTN | MBNL1-AS1 | 0.636268224 | 3.89E-48 | postive |
| FLNA | MBNL1-AS1 | 0.704300803 | 5.47E-63 | postive |
| TLN1 | MBNL1-AS1 | 0.494133993 | 9.45E-27 | postive |
| ACTN1 | MBNL1-AS1 | 0.492147185 | 1.61E-26 | postive |
| DSTN | AP003071.4 | 0.549231894 | 7.72E-34 | postive |
| FLNA | AP003071.4 | 0.645793267 | 5.47E-50 | postive |
| TLN1 | AP003071.4 | 0.447629446 | 1.08E-21 | postive |
| ACTN1 | AP003071.4 | 0.477054474 | 8.38E-25 | postive |
| NDUFA11 | H1-10-AS1 | 0.555694808 | 9.27E-35 | postive |
| NDUFS1 | AL132800.1 | 0.431496744 | 4.10E-20 | postive |
| NUBPL | AL132800.1 | 0.508808999 | 1.63E-28 | postive |
| NCKAP1 | AL132800.1 | 0.401358622 | 2.23E-17 | postive |
| CD2AP | AL132800.1 | 0.412634738 | 2.28E-18 | postive |
| CD2AP | AC005522.1 | 0.401084124 | 2.35E-17 | postive |
| FLNA | AC107959.1 | 0.40163424 | 2.11E-17 | postive |
| TLN1 | AC107959.1 | 0.454041267 | 2.40E-22 | postive |
| DSTN | AC036108.3 | 0.646258452 | 4.43E-50 | postive |
| FLNA | AC036108.3 | 0.708557097 | 4.64E-64 | postive |
| TLN1 | AC036108.3 | 0.51102729 | 8.67E-29 | postive |
| ACTN1 | AC036108.3 | 0.432113374 | 3.58E-20 | postive |
| CD2AP | AC024361.1 | 0.442878226 | 3.21E-21 | postive |
| CD2AP | AC068189.2 | 0.414816539 | 1.45E-18 | postive |
| CD2AP | AC022272.1 | 0.470662208 | 4.21E-24 | postive |
| NUBPL | AC022364.1 | 0.429016936 | 7.06E-20 | postive |
| NCKAP1 | AC022364.1 | 0.451143137 | 4.75E-22 | postive |
| CD2AP | AC009087.1 | 0.418750872 | 6.36E-19 | postive |
| CD2AP | AC138956.2 | 0.5031278 | 8.03E-28 | postive |
| ACTB | AP000866.5 | -0.427512929 | 9.79E-20 | negative |
| NDUFA11 | AC010331.1 | 0.465331567 | 1.58E-23 | postive |
| OXSM | AC008763.1 | 0.409653062 | 4.20E-18 | postive |
| DSTN | ZNF710-AS1 | 0.423952181 | 2.11E-19 | postive |
| FLNA | ZNF710-AS1 | 0.461486461 | 4.03E-23 | postive |
| TLN1 | ZNF710-AS1 | 0.458462383 | 8.37E-23 | postive |
| TLN1 | AL513165.1 | 0.404662991 | 1.15E-17 | postive |
| FLNA | AC013553.3 | 0.4948682 | 7.75E-27 | postive |
| TLN1 | AC013553.3 | 0.479152358 | 4.89E-25 | postive |
| SLC7A11 | AC093484.4 | 0.420892907 | 4.05E-19 | postive |
| ACTN1 | SPINT1-AS1 | -0.413591628 | 1.87E-18 | negative |
| CD2AP | Z98884.2 | 0.413442861 | 1.93E-18 | postive |
| SLC7A11 | AC069277.1 | 0.645442876 | 6.42E-50 | postive |
| NDUFA11 | AC010487.1 | 0.541781613 | 8.39E-33 | postive |
| CD2AP | AC131025.3 | 0.425022629 | 1.68E-19 | postive |
| TLN1 | COL4A2-AS1 | 0.514853782 | 2.89E-29 | postive |
| CD2AP | AC009121.2 | 0.451694911 | 4.17E-22 | postive |
| SLC7A11 | DUBR | 0.52795789 | 6.01E-31 | postive |
| TLN1 | DUBR | 0.415275201 | 1.32E-18 | postive |
| NDUFA11 | AC022966.2 | 0.491602527 | 1.87E-26 | postive |
| CD2AP | AC016405.1 | 0.423789976 | 2.18E-19 | postive |
| TLN1 | LINC01119 | 0.464232568 | 2.07E-23 | postive |
| CD2AP | LINC00630 | 0.46609121 | 1.31E-23 | postive |
| CD2AP | AC097641.2 | 0.456531009 | 1.33E-22 | postive |
| CD2AP | NUTM2B-AS1 | 0.444568927 | 2.18E-21 | postive |
| NUBPL | AC234917.3 | 0.415938332 | 1.15E-18 | postive |
| LRPPRC | AC234917.3 | 0.421850523 | 3.30E-19 | postive |
| NDUFA11 | AL021707.8 | 0.502974925 | 8.38E-28 | postive |
| CD2AP | AP001628.1 | 0.464979236 | 1.72E-23 | postive |
| NDUFA11 | AC067852.5 | 0.506230183 | 3.37E-28 | postive |
| SLC7A11 | AL355102.4 | 0.521308905 | 4.38E-30 | postive |
| SLC7A11 | STARD13-AS | 0.667179688 | 2.13E-54 | postive |
| CD2AP | KDM4A-AS1 | 0.481615846 | 2.59E-25 | postive |
| SLC7A11 | AC004846.2 | 0.651647625 | 3.69E-51 | postive |
| FLNA | AC092376.3 | 0.412933158 | 2.14E-18 | postive |
| TLN1 | AC092376.3 | 0.401446365 | 2.19E-17 | postive |
| NDUFA11 | AC016773.1 | 0.439466095 | 6.96E-21 | postive |
| SLC7A11 | RERG-IT1 | 0.623245519 | 1.05E-45 | postive |
| NDUFA11 | SNHG9 | 0.473049746 | 2.31E-24 | postive |
| IQGAP1 | SNHG9 | -0.426535736 | 1.21E-19 | negative |
| NDUFA11 | AL035587.2 | 0.526213745 | 1.02E-30 | postive |
| MYL6 | AL035587.2 | 0.402337441 | 1.84E-17 | postive |
| SLC7A11 | SGMS1-AS1 | 0.465787919 | 1.41E-23 | postive |
| SLC7A11 | AGAP1-IT1 | 0.465860664 | 1.39E-23 | postive |
| NDUFA11 | AL354836.1 | 0.464314004 | 2.02E-23 | postive |
| SLC7A11 | AC093620.1 | 0.409318918 | 4.49E-18 | postive |
| SLC7A11 | RASA3-IT1 | 0.612367749 | 9.18E-44 | postive |
| SLC7A11 | AL590428.1 | 0.412711814 | 2.24E-18 | postive |
| ACTB | AL590428.1 | 0.433592964 | 2.58E-20 | postive |
| FLNA | AL590428.1 | 0.434034692 | 2.34E-20 | postive |
| TLN1 | AL590428.1 | 0.548636691 | 9.36E-34 | postive |
| ACTN1 | AL590428.1 | 0.437874963 | 9.96E-21 | postive |
| SLC7A11 | FENDRR | 0.525475384 | 1.27E-30 | postive |
| FLNA | FENDRR | 0.515875123 | 2.15E-29 | postive |
| TLN1 | FENDRR | 0.501398851 | 1.30E-27 | postive |
| NDUFA11 | AC145285.6 | 0.454043094 | 2.40E-22 | postive |
| NDUFS1 | AC107027.3 | 0.448688299 | 8.42E-22 | postive |
| CD2AP | ELOA-AS1 | 0.408431278 | 5.38E-18 | postive |
| SLC7A11 | AC079921.1 | 0.697501068 | 2.57E-61 | postive |
| CD2AP | AL391839.2 | 0.450057509 | 6.12E-22 | postive |
| CD2AP | AC092611.1 | 0.407031178 | 7.15E-18 | postive |
| NCKAP1 | C2orf27A | 0.471457666 | 3.45E-24 | postive |
| CD2AP | AC090579.1 | 0.418456838 | 6.77E-19 | postive |
| CD2AP | AC078983.1 | 0.407238645 | 6.86E-18 | postive |
| SLC7A11 | AC007922.2 | 0.454332721 | 2.24E-22 | postive |
| NDUFA11 | AC132872.2 | 0.42779117 | 9.21E-20 | postive |
| DSTN | AL136084.2 | 0.546508692 | 1.86E-33 | postive |
| FLNA | AL136084.2 | 0.592924806 | 1.79E-40 | postive |
| TLN1 | AL136084.2 | 0.416770446 | 9.64E-19 | postive |
| ACTN1 | AL136084.2 | 0.466870646 | 1.08E-23 | postive |
| PRDX1 | LINC02762 | 0.400867006 | 2.46E-17 | postive |
| SLC7A11 | LAMC1-AS1 | 0.489480354 | 3.29E-26 | postive |
| MYL6 | ZSCAN16-AS1 | 0.517617282 | 1.29E-29 | postive |
| CD2AP | AF117829.1 | 0.411959458 | 2.61E-18 | postive |
| MYH10 | POLH-AS1 | 0.534245289 | 8.82E-32 | postive |
| CD2AP | AC005014.4 | 0.492196782 | 1.59E-26 | postive |
| CD2AP | AC091906.1 | 0.443072098 | 3.07E-21 | postive |
| SLC7A11 | ITGA9-AS1 | 0.470713081 | 4.16E-24 | postive |
| SLC7A11 | AC040904.1 | 0.622050554 | 1.73E-45 | postive |
| SLC7A11 | AC069549.1 | 0.643919679 | 1.28E-49 | postive |
| SLC7A11 | AC007620.3 | 0.519293272 | 7.93E-30 | postive |
| TLN1 | AC008050.1 | 0.417431185 | 8.39E-19 | postive |
| CD2AP | SH3TC2-DT | 0.400153046 | 2.83E-17 | postive |
| SLC7A11 | AC087286.1 | 0.521073297 | 4.69E-30 | postive |
| FLNA | RASSF8-AS1 | 0.409534659 | 4.30E-18 | postive |
| MYH10 | RASSF8-AS1 | 0.471384404 | 3.51E-24 | postive |
| TLN1 | RASSF8-AS1 | 0.530462754 | 2.81E-31 | postive |
| ACTN1 | RASSF8-AS1 | 0.452841189 | 3.19E-22 | postive |
| SLC7A11 | AC100810.3 | 0.532424518 | 1.54E-31 | postive |
| CD2AP | CRTC3-AS1 | 0.50809876 | 1.99E-28 | postive |
| CD2AP | AC024361.3 | 0.456944078 | 1.20E-22 | postive |
| CD2AP | AL021368.2 | 0.439449703 | 6.99E-21 | postive |
| DSTN | AC106897.1 | 0.42944697 | 6.43E-20 | postive |
| FLNA | AC106897.1 | 0.556391259 | 7.36E-35 | postive |
| TLN1 | AC106897.1 | 0.47874963 | 5.43E-25 | postive |
| ACTN1 | AC106897.1 | 0.463817906 | 2.29E-23 | postive |
| NDUFA11 | AL163051.1 | 0.44209652 | 3.84E-21 | postive |
| CD2AP | AC011405.1 | 0.424315617 | 1.95E-19 | postive |
| CD2AP | LINC02569 | 0.426905506 | 1.12E-19 | postive |
| SLC7A11 | AC008569.2 | 0.485384134 | 9.70E-26 | postive |
| CD2AP | AC009318.3 | 0.469209439 | 6.05E-24 | postive |
| NDUFA11 | AC008608.2 | 0.423546318 | 2.30E-19 | postive |
| ACTB | AC002480.1 | 0.433395338 | 2.70E-20 | postive |
| SLC7A11 | AL133476.1 | 0.651089514 | 4.78E-51 | postive |
| TLN1 | AL133476.1 | 0.434333151 | 2.19E-20 | postive |
| SLC7A11 | LRRC8C-DT | 0.415929985 | 1.15E-18 | postive |
| CD2AP | MORC2-AS1 | 0.422070304 | 3.15E-19 | postive |
| SLC7A11 | LINC01697 | 0.571758237 | 3.90E-37 | postive |
| DSTN | MIR1-1HG-AS1 | 0.628185648 | 1.29E-46 | postive |
| FLNA | MIR1-1HG-AS1 | 0.715759098 | 6.44E-66 | postive |
| TLN1 | MIR1-1HG-AS1 | 0.496419918 | 5.08E-27 | postive |
| ACTN1 | MIR1-1HG-AS1 | 0.435622701 | 1.65E-20 | postive |
| NDUFA11 | AL645940.1 | 0.466459974 | 1.20E-23 | postive |
| CD2AP | SDK1-AS1 | 0.436053573 | 1.50E-20 | postive |
| NDUFA11 | AC024060.2 | 0.558707941 | 3.40E-35 | postive |
| SLC7A11 | AL157394.3 | 0.471084484 | 3.79E-24 | postive |
| NDUFA11 | AC104785.1 | 0.431026843 | 4.55E-20 | postive |
| SLC7A11 | AC016831.1 | 0.596487157 | 4.65E-41 | postive |
| SLC7A11 | AC019183.1 | 0.52810567 | 5.75E-31 | postive |
| ACTN1 | AC134312.5 | 0.430345152 | 5.28E-20 | postive |
| SLC7A11 | AC138207.9 | 0.730261963 | 7.71E-70 | postive |
| ACTN1 | ACTN1-AS1 | 0.531329337 | 2.16E-31 | postive |
| SLC7A11 | AL121957.1 | 0.419954194 | 4.94E-19 | postive |
| NDUFA11 | AP003419.3 | 0.563574781 | 6.58E-36 | postive |
| FLNA | THCAT158 | 0.427265756 | 1.03E-19 | postive |
| TLN1 | THCAT158 | 0.448260806 | 9.30E-22 | postive |
| NUBPL | AC121338.2 | 0.435424336 | 1.72E-20 | postive |
| TLN2 | AC121338.2 | 0.430671729 | 4.92E-20 | postive |
| FLNA | AC106881.1 | 0.43330369 | 2.76E-20 | postive |
| MYH10 | AC106881.1 | 0.545762395 | 2.36E-33 | postive |
| ACTN1 | AP000695.1 | 0.41956095 | 5.36E-19 | postive |
| SLC7A11 | ZFPM2-AS1 | 0.404783646 | 1.12E-17 | postive |
| CD2AP | AP001469.2 | 0.403576484 | 1.43E-17 | postive |
| SLC7A11 | AC023825.2 | 0.662297826 | 2.33E-53 | postive |
| CD2AP | AC018521.6 | 0.426833123 | 1.13E-19 | postive |
| NDUFA11 | AL390719.2 | 0.540818283 | 1.14E-32 | postive |
| ACTB | AL390719.2 | -0.416370658 | 1.05E-18 | negative |
| OXSM | AC112220.2 | 0.516354406 | 1.87E-29 | postive |
| CD2AP | AC006141.1 | 0.445398956 | 1.80E-21 | postive |
| CD2AP | AC253576.2 | 0.410100946 | 3.83E-18 | postive |
| CD2AP | MIR2052HG | 0.403061364 | 1.59E-17 | postive |
| CD2AP | AC026704.1 | 0.408586661 | 5.21E-18 | postive |
| DSTN | ACTA2-AS1 | 0.600594152 | 9.60E-42 | postive |
| FLNA | ACTA2-AS1 | 0.682558871 | 8.41E-58 | postive |
| TLN1 | ACTA2-AS1 | 0.502159203 | 1.05E-27 | postive |
| ACTN1 | ACTA2-AS1 | 0.4955665 | 6.41E-27 | postive |
| CD2AP | AC108052.1 | 0.448178188 | 9.48E-22 | postive |
| SLC7A11 | AC093515.1 | 0.454769391 | 2.02E-22 | postive |
| DSTN | LINC02884 | 0.419560793 | 5.36E-19 | postive |
| FLNA | LINC02884 | 0.483903742 | 1.43E-25 | postive |
| TLN1 | LINC02884 | 0.403048224 | 1.59E-17 | postive |
| CD2AP | AC016394.2 | 0.420934972 | 4.01E-19 | postive |
| CAPZB | LINC02606 | 0.431683158 | 3.94E-20 | postive |
| FLNA | LINC02606 | 0.510510309 | 1.00E-28 | postive |
| TLN1 | LINC02606 | 0.565026005 | 4.01E-36 | postive |
| NDUFA11 | AC007292.1 | 0.536888445 | 3.89E-32 | postive |
| CD2AP | AP001178.1 | 0.439783135 | 6.48E-21 | postive |
| SLC7A11 | LINC02595 | 0.597573274 | 3.07E-41 | postive |
| CD2AP | AC024560.4 | 0.494972606 | 7.53E-27 | postive |
| SLC7A11 | RBMS3-AS2 | 0.69956359 | 8.09E-62 | postive |
| CD2AP | AL138756.1 | 0.503622822 | 7.00E-28 | postive |
| CD2AP | LINC01409 | 0.445750819 | 1.66E-21 | postive |
| FLNA | LINC02693 | 0.403097848 | 1.58E-17 | postive |
| TLN1 | LINC02693 | 0.437665563 | 1.04E-20 | postive |
| ACTN1 | LINC02693 | 0.409479934 | 4.35E-18 | postive |
| NDUFA11 | AC015912.3 | 0.43512666 | 1.84E-20 | postive |
| CD2AP | AL139099.2 | 0.489167907 | 3.57E-26 | postive |
| SLC7A11 | LINC00308 | 0.622624543 | 1.36E-45 | postive |
| CD2AP | MKLN1-AS | 0.425741057 | 1.44E-19 | postive |
| NDUFS1 | AC073254.1 | 0.517356188 | 1.40E-29 | postive |
| NCKAP1 | AC073254.1 | 0.416372013 | 1.05E-18 | postive |
| SLC7A11 | AC005740.4 | 0.508798843 | 1.63E-28 | postive |
| PRDX1 | AC092115.3 | 0.417256756 | 8.71E-19 | postive |
| SLC7A11 | AC092301.1 | 0.41901918 | 6.01E-19 | postive |
| TLN2 | AC091179.1 | 0.607066831 | 7.63E-43 | postive |
| CD2AP | AL354993.2 | 0.531857952 | 1.84E-31 | postive |
| CD2AP | AC008507.2 | 0.436276009 | 1.42E-20 | postive |
| SLC7A11 | AC090559.1 | 0.4359327 | 1.54E-20 | postive |
| ACTB | AC090559.1 | 0.439560586 | 6.82E-21 | postive |
| FLNA | AC090559.1 | 0.466042942 | 1.32E-23 | postive |
| TLN1 | AC090559.1 | 0.600843746 | 8.72E-42 | postive |
| ACTN1 | AC090559.1 | 0.438100529 | 9.47E-21 | postive |
| NDUFA11 | AC046143.2 | 0.407306493 | 6.76E-18 | postive |
| TLN1 | AC104938.1 | 0.46929024 | 5.93E-24 | postive |
| NDUFA11 | AC008764.6 | 0.51676153 | 1.66E-29 | postive |
| IQGAP1 | AL450326.1 | 0.43180675 | 3.83E-20 | postive |
| MYH9 | AL450326.1 | 0.407627719 | 6.34E-18 | postive |
| TLN1 | AL450326.1 | 0.535942104 | 5.22E-32 | postive |
| SLC7A11 | AC090907.3 | 0.525249698 | 1.36E-30 | postive |
| SLC7A11 | AL138995.1 | 0.418394215 | 6.86E-19 | postive |
| TLN1 | AL138995.1 | 0.435185082 | 1.82E-20 | postive |
| SLC7A11 | AC005730.3 | 0.454699471 | 2.05E-22 | postive |
| CD2AP | AC048382.1 | 0.497219949 | 4.09E-27 | postive |
| CD2AP | AC022137.3 | 0.45270192 | 3.29E-22 | postive |
| NDUFA11 | MIR200CHG | 0.493164175 | 1.23E-26 | postive |
| MYH10 | AC080038.1 | 0.450782266 | 5.17E-22 | postive |
| CD2AP | DLEU1 | 0.443546053 | 2.76E-21 | postive |
| NDUFA11 | AC009065.4 | 0.643854716 | 1.32E-49 | postive |
| SLC7A11 | AC100763.1 | 0.412266856 | 2.45E-18 | postive |
| TLN1 | LINC01150 | 0.459314124 | 6.82E-23 | postive |
| NDUFA11 | AC009148.1 | 0.440204398 | 5.89E-21 | postive |
| CD2AP | NR2F2-AS1 | 0.416183584 | 1.09E-18 | postive |
| SLC7A11 | KIF26B-AS1 | 0.462005195 | 3.56E-23 | postive |
| SLC7A11 | WWTR1-IT1 | 0.707492492 | 8.63E-64 | postive |
| CD2AP | AC021491.4 | 0.430744689 | 4.84E-20 | postive |
| CD2AP | UBL7-AS1 | 0.456079366 | 1.48E-22 | postive |
| NDUFA11 | AL590101.1 | 0.403468256 | 1.46E-17 | postive |
| CD2AP | AC007528.1 | 0.401714399 | 2.08E-17 | postive |
| CD2AP | AC211433.1 | 0.415790688 | 1.18E-18 | postive |
| CD2AP | AC026124.2 | 0.414044037 | 1.70E-18 | postive |
| CD2AP | AC007681.1 | 0.412586857 | 2.30E-18 | postive |
| CD2AP | AC011442.1 | 0.430407618 | 5.21E-20 | postive |
| SLC7A11 | MIR4435-2HG | 0.486356583 | 7.51E-26 | postive |
| NDUFA11 | SNHG25 | 0.610764808 | 1.75E-43 | postive |
| NDUFA11 | AC011462.4 | 0.48361513 | 1.54E-25 | postive |
| TLN1 | LINC01852 | 0.431313346 | 4.27E-20 | postive |
| TLN2 | LINC01852 | 0.433961249 | 2.38E-20 | postive |
| CD2AP | AL080317.1 | 0.442013203 | 3.91E-21 | postive |
| CD2AP | AC073349.4 | 0.443484551 | 2.80E-21 | postive |
| CD2AP | AL590652.1 | 0.424417834 | 1.91E-19 | postive |
| CD2AP | AC005253.1 | 0.418292297 | 7.01E-19 | postive |
| NDUFS1 | AC015922.2 | 0.426157071 | 1.31E-19 | postive |
| NCKAP1 | AC015922.2 | 0.432049144 | 3.63E-20 | postive |
| CD2AP | AC015922.2 | 0.411667608 | 2.78E-18 | postive |
| NDUFA11 | RAB11B-AS1 | 0.582179 | 9.52E-39 | postive |
| CD2AP | FIRRE | 0.419395437 | 5.55E-19 | postive |
| DSTN | HAND2-AS1 | 0.653770866 | 1.37E-51 | postive |
| FLNA | HAND2-AS1 | 0.722681555 | 9.29E-68 | postive |
| TLN1 | HAND2-AS1 | 0.502445949 | 9.71E-28 | postive |
| ACTN1 | HAND2-AS1 | 0.494458176 | 8.66E-27 | postive |
| SLC7A11 | LINC01484 | 0.571778263 | 3.87E-37 | postive |
| CD2AP | AF129075.3 | 0.425314964 | 1.57E-19 | postive |
| MYH10 | PCAT19 | 0.435927832 | 1.54E-20 | postive |
| DSTN | LINC02829 | 0.478295446 | 6.10E-25 | postive |
| FLNA | LINC02829 | 0.542606297 | 6.46E-33 | postive |
| FLNA | MAGI2-AS3 | 0.4398564 | 6.38E-21 | postive |
| TLN1 | MAGI2-AS3 | 0.469811079 | 5.21E-24 | postive |
| SLC7A11 | AC011092.3 | 0.412492309 | 2.34E-18 | postive |
| CD2AP | AP001630.1 | 0.438335902 | 8.98E-21 | postive |
| DSTN | LINC01936 | 0.438197809 | 9.26E-21 | postive |
| FLNA | LINC01936 | 0.465278058 | 1.60E-23 | postive |
| TLN1 | AP001471.1 | 0.45689465 | 1.22E-22 | postive |
| ACTN1 | AP001471.1 | 0.40885281 | 4.94E-18 | postive |
| DSTN | AC027449.1 | 0.598610581 | 2.06E-41 | postive |
| FLNA | AC027449.1 | 0.617474241 | 1.15E-44 | postive |
| TLN1 | AC027449.1 | 0.421121801 | 3.85E-19 | postive |
| ACTN1 | AC027449.1 | 0.413145614 | 2.05E-18 | postive |
| SLC7A11 | AL133371.2 | 0.50864683 | 1.71E-28 | postive |
| TLN1 | AL133371.2 | 0.4592851 | 6.86E-23 | postive |
| DSTN | AC005180.2 | 0.687193421 | 7.20E-59 | postive |
| FLNA | AC005180.2 | 0.75420321 | 6.63E-77 | postive |
| TLN1 | AC005180.2 | 0.503068271 | 8.17E-28 | postive |
| ACTN1 | AC005180.2 | 0.48448401 | 1.23E-25 | postive |
| NDUFA11 | AL691432.4 | 0.461777845 | 3.76E-23 | postive |
| NDUFA11 | LINC02804 | 0.415212136 | 1.33E-18 | postive |
| NDUFA11 | ZFAS1 | 0.498353256 | 3.00E-27 | postive |
| CD2AP | AC100821.2 | 0.402792819 | 1.68E-17 | postive |
| SLC7A11 | AL139042.1 | 0.602606982 | 4.40E-42 | postive |
| OXSM | ENTPD3-AS1 | 0.413014069 | 2.10E-18 | postive |
| CD2AP | AC010300.1 | 0.418993999 | 6.04E-19 | postive |
| CD2AP | AC092279.1 | 0.473952543 | 1.84E-24 | postive |
| CD2AP | AC005379.1 | 0.46288299 | 2.87E-23 | postive |
| CD2AP | AC008883.1 | 0.410106979 | 3.82E-18 | postive |
| CD2AP | AC010976.1 | 0.46157455 | 3.95E-23 | postive |
| SLC7A11 | AC093520.1 | 0.400912462 | 2.44E-17 | postive |
| CD2AP | FAM87B | 0.412135932 | 2.52E-18 | postive |
| CD2AP | AC037487.1 | 0.432014864 | 3.66E-20 | postive |
| DSTN | CADM3-AS1 | 0.44360505 | 2.72E-21 | postive |
| FLNA | CADM3-AS1 | 0.449650025 | 6.73E-22 | postive |
| FLNA | LINC00163 | 0.415882844 | 1.16E-18 | postive |
| TLN1 | LINC00163 | 0.40737986 | 6.66E-18 | postive |
| SLC7A11 | LINC-PINT | 0.465312189 | 1.59E-23 | postive |
| CD2AP | AC107419.1 | 0.40371432 | 1.39E-17 | postive |
| DSTN | AC022893.1 | 0.468148796 | 7.87E-24 | postive |
| FLNA | AC022893.1 | 0.544328275 | 3.73E-33 | postive |
| TLN1 | AC022893.1 | 0.451455427 | 4.41E-22 | postive |
| NDUFA11 | AC002398.1 | 0.442040101 | 3.89E-21 | postive |
| CD2AP | LINC00628 | 0.431276979 | 4.31E-20 | postive |
| SLC7A11 | AC083870.1 | 0.556508492 | 7.08E-35 | postive |
| ACTB | AL135999.1 | -0.403238967 | 1.53E-17 | negative |
| CD2AP | AC015871.6 | 0.434951434 | 1.91E-20 | postive |
| ACTN1 | SCAT1 | 0.45531955 | 1.77E-22 | postive |
| CD2AP | FARP1-AS1 | 0.401073919 | 2.36E-17 | postive |
| CD2AP | UBE2Q1-AS1 | 0.470104716 | 4.84E-24 | postive |
| CD2AP | AL732509.1 | 0.415890075 | 1.16E-18 | postive |
| FLNA | LINC02202 | 0.452149732 | 3.75E-22 | postive |
| NDUFS1 | SMARCA5-AS1 | 0.423649757 | 2.25E-19 | postive |
| NCKAP1 | SMARCA5-AS1 | 0.413201222 | 2.02E-18 | postive |
| LRPPRC | SMARCA5-AS1 | 0.415958121 | 1.14E-18 | postive |
| IQGAP1 | SMARCA5-AS1 | 0.413568894 | 1.88E-18 | postive |
| SLC7A11 | AC007319.1 | 0.476139131 | 1.06E-24 | postive |
| SLC7A11 | AC019080.5 | 0.402848482 | 1.66E-17 | postive |
| NDUFA11 | AC138230.1 | 0.441395042 | 4.50E-21 | postive |
| SLC7A11 | MIR223HG | 0.5068184 | 2.86E-28 | postive |
| SLC7A11 | AC011933.3 | 0.400747687 | 2.52E-17 | postive |
| SLC7A11 | AC108449.2 | 0.410088659 | 3.84E-18 | postive |
| NUBPL | AC108449.2 | 0.436798211 | 1.27E-20 | postive |
| CD2AP | AC108449.2 | 0.457721655 | 9.99E-23 | postive |
| NDUFA11 | HEIH | 0.434411433 | 2.16E-20 | postive |
| NDUFS1 | AC012063.1 | 0.403871327 | 1.35E-17 | postive |
| NUBPL | AC012063.1 | 0.417244222 | 8.73E-19 | postive |
| CD2AP | AL450344.3 | 0.435056237 | 1.87E-20 | postive |
| CD2AP | AC004596.1 | 0.442952442 | 3.16E-21 | postive |
| SLC7A11 | AC010525.1 | 0.456022167 | 1.50E-22 | postive |
| SLC7A11 | HMGA2-AS1 | 0.667569388 | 1.76E-54 | postive |
| CD2AP | AC092802.2 | 0.495101063 | 7.27E-27 | postive |
| SLC7A11 | AL137779.1 | 0.486533551 | 7.17E-26 | postive |
| CD2AP | LINC02520 | 0.426560447 | 1.20E-19 | postive |
| SLC7A11 | C2-AS1 | 0.417916438 | 7.58E-19 | postive |
| SLC7A11 | LUARIS | 0.584361858 | 4.30E-39 | postive |
| NCKAP1 | MIR4713HG | 0.460699321 | 4.88E-23 | postive |
| IQGAP1 | MIR4713HG | 0.411663184 | 2.78E-18 | postive |
| CD2AP | AC078852.2 | 0.407979443 | 5.90E-18 | postive |
| CD2AP | AC093752.2 | 0.442283115 | 3.68E-21 | postive |
| CD2AP | AC015923.1 | 0.438575864 | 8.51E-21 | postive |
| SLC7A11 | AC018752.1 | 0.453010171 | 3.06E-22 | postive |
| NUBPL | AC018752.1 | 0.420448784 | 4.44E-19 | postive |
| SLC7A11 | AC108463.2 | 0.456056773 | 1.49E-22 | postive |
| SLC7A11 | AC138393.3 | 0.426277209 | 1.28E-19 | postive |
| DSTN | AC079313.1 | 0.418345286 | 6.93E-19 | postive |
| FLNA | AC079313.1 | 0.517397691 | 1.38E-29 | postive |
| SLC7A11 | NXT1-AS1 | 0.515931994 | 2.11E-29 | postive |
| NDUFA11 | AC087289.2 | 0.440352309 | 5.70E-21 | postive |
| ACTB | AL353807.5 | 0.402390081 | 1.82E-17 | postive |
| SLC7A11 | AC087854.1 | 0.540481193 | 1.26E-32 | postive |
| CD2AP | SNHG14 | 0.49228783 | 1.55E-26 | postive |
| SLC7A11 | AP003171.1 | 0.639917623 | 7.74E-49 | postive |
| NUBPL | LINC01184 | 0.428315442 | 8.22E-20 | postive |
| SLC7A11 | AC006159.1 | 0.61489704 | 3.29E-44 | postive |
| NUBPL | RAP2C-AS1 | 0.428685176 | 7.59E-20 | postive |
| IQGAP1 | RAP2C-AS1 | 0.413398858 | 1.94E-18 | postive |
| RPN1 | AC137695.3 | 0.454873767 | 1.97E-22 | postive |
| SLC7A11 | AC108463.3 | 0.546403131 | 1.92E-33 | postive |
| CD2AP | AC145146.1 | 0.409438499 | 4.38E-18 | postive |
| CD2AP | AC020558.6 | 0.448211689 | 9.41E-22 | postive |
| CD2AP | AL080317.2 | 0.458585025 | 8.12E-23 | postive |
| SLC7A11 | SH3RF3-AS1 | 0.453525187 | 2.71E-22 | postive |
| FLNA | SH3RF3-AS1 | 0.430530851 | 5.07E-20 | postive |
| TLN1 | SH3RF3-AS1 | 0.618788991 | 6.68E-45 | postive |
| SLC7A11 | AC100830.1 | 0.440529415 | 5.48E-21 | postive |
| CD2AP | AL592435.1 | 0.406365713 | 8.18E-18 | postive |
| CD2AP | AC092794.1 | 0.401448251 | 2.19E-17 | postive |
| SLC7A11 | MAP3K20-AS1 | 0.616110631 | 2.01E-44 | postive |
| SLC7A11 | JARID2-AS1 | 0.475342549 | 1.30E-24 | postive |
| CD2AP | AC073046.1 | 0.424156165 | 2.02E-19 | postive |
| SLC7A11 | AL161891.1 | 0.433493716 | 2.64E-20 | postive |
| NDUFS1 | AL161891.1 | 0.408170029 | 5.68E-18 | postive |
| LRPPRC | AL161891.1 | 0.421062557 | 3.90E-19 | postive |
| SLC7A11 | SLC7A11-AS1 | 0.753179807 | 1.38E-76 | postive |
| CD2AP | AL117344.2 | 0.469839253 | 5.17E-24 | postive |
| TLN1 | AC013565.1 | 0.406763475 | 7.55E-18 | postive |
| SLC7A11 | AC022154.1 | 0.453784739 | 2.55E-22 | postive |
| DSTN | AC005180.1 | 0.668359032 | 1.19E-54 | postive |
| FLNA | AC005180.1 | 0.749455425 | 1.93E-75 | postive |
| TLN1 | AC005180.1 | 0.498908482 | 2.58E-27 | postive |
| ACTN1 | AC005180.1 | 0.488156117 | 4.67E-26 | postive |
| SLC7A11 | AL031429.2 | 0.489529334 | 3.25E-26 | postive |
| SLC7A11 | DLEU2 | 0.440418495 | 5.61E-21 | postive |
| FLNA | AP001189.3 | 0.432591255 | 3.22E-20 | postive |
| TLN1 | AP001189.3 | 0.442151318 | 3.79E-21 | postive |
| ACTN1 | AP001189.3 | 0.403772088 | 1.38E-17 | postive |
| CD2AP | AC073896.2 | 0.470248716 | 4.67E-24 | postive |
| CD2AP | AC034102.8 | 0.459332338 | 6.79E-23 | postive |
| SLC7A11 | AC008443.2 | 0.403685677 | 1.40E-17 | postive |
| ACTB | AC007877.1 | 0.431787784 | 3.85E-20 | postive |
| FLNA | AC007877.1 | 0.430550202 | 5.05E-20 | postive |
| MYL6 | AC007877.1 | 0.42772009 | 9.36E-20 | postive |
| TLN1 | AC007877.1 | 0.420576542 | 4.33E-19 | postive |
| CD2AP | GHRLOS | 0.429162634 | 6.84E-20 | postive |
| CD2AP | AC006270.1 | 0.435357964 | 1.75E-20 | postive |
| SLC7A11 | LINC01203 | 0.504896348 | 4.90E-28 | postive |
| CD2AP | AC004039.1 | 0.413883028 | 1.76E-18 | postive |
| SLC7A11 | MIR222HG | 0.486862206 | 6.58E-26 | postive |
| ACTB | VIM-AS1 | 0.487040136 | 6.28E-26 | postive |
| ACTN1 | VIM-AS1 | 0.454042913 | 2.40E-22 | postive |
| SLC7A11 | AC037487.2 | 0.570243743 | 6.62E-37 | postive |
| TLN2 | AC124798.1 | 0.481005663 | 3.04E-25 | postive |
| CD2AP | AC106037.2 | 0.418810508 | 6.28E-19 | postive |
| CD2AP | AC008119.1 | 0.433011473 | 2.94E-20 | postive |
| CD2AP | AL136221.1 | 0.435031724 | 1.88E-20 | postive |
| CD2AP | AC024075.3 | 0.429176617 | 6.82E-20 | postive |
| CD2AP | AC011477.2 | 0.479533865 | 4.44E-25 | postive |
| CD2AP | PCBP2-OT1 | 0.417527711 | 8.23E-19 | postive |
| SLC7A11 | AL355102.1 | 0.718335903 | 1.35E-66 | postive |
| SLC7A11 | AC138207.4 | 0.677865758 | 9.68E-57 | postive |
| MYH10 | OXCT1-AS1 | 0.430209348 | 5.44E-20 | postive |
| SLC7A11 | AC104170.1 | 0.478504235 | 5.78E-25 | postive |
| SLC7A11 | IPO9-AS1 | 0.408611256 | 5.19E-18 | postive |
| CD2AP | LINC01876 | 0.480674236 | 3.31E-25 | postive |
| SLC7A11 | AL162724.2 | 0.681771994 | 1.27E-57 | postive |
| DSTN | LINC02489 | 0.556070163 | 8.19E-35 | postive |
| FLNA | LINC02489 | 0.625021881 | 4.96E-46 | postive |
| TLN1 | LINC02489 | 0.421828209 | 3.32E-19 | postive |
| MYH10 | AC090152.1 | 0.614550694 | 3.79E-44 | postive |
| DSTN | AC103740.1 | 0.523671747 | 2.17E-30 | postive |
| FLNA | AC103740.1 | 0.577196382 | 5.71E-38 | postive |
| TLN1 | AC103740.1 | 0.452516389 | 3.44E-22 | postive |
| SLC7A11 | GNG12-AS1 | 0.65841695 | 1.51E-52 | postive |
| NDUFS1 | AC024075.1 | 0.41307506 | 2.08E-18 | postive |
| CD2AP | FOXP1-IT1 | 0.415081922 | 1.37E-18 | postive |
| CD2AP | AC009090.6 | 0.481867215 | 2.43E-25 | postive |
| DSTN | ADAMTS9-AS2 | 0.488948341 | 3.79E-26 | postive |
| FLNA | ADAMTS9-AS2 | 0.583608785 | 5.66E-39 | postive |
| TLN1 | ADAMTS9-AS2 | 0.472429398 | 2.70E-24 | postive |
| NDUFA11 | MHENCR | 0.549874093 | 6.26E-34 | postive |
| IQGAP1 | MHENCR | -0.400404679 | 2.69E-17 | negative |
| NDUFA11 | AL513320.1 | 0.503159958 | 7.96E-28 | postive |
| CD2AP | AC009686.1 | 0.432005075 | 3.67E-20 | postive |
| DSTN | AC068506.1 | 0.478728474 | 5.46E-25 | postive |
| FLNA | AC068506.1 | 0.569182558 | 9.57E-37 | postive |
| TLN1 | AC068506.1 | 0.405662818 | 9.43E-18 | postive |
| ACTN1 | AC068506.1 | 0.433634993 | 2.56E-20 | postive |
| NDUFA11 | AL021707.6 | 0.546316566 | 1.98E-33 | postive |
| TLN1 | AC019254.1 | 0.432382531 | 3.38E-20 | postive |
| SLC7A11 | AC215522.2 | 0.524555325 | 1.67E-30 | postive |
| MYH10 | RAMP2-AS1 | 0.401518804 | 2.16E-17 | postive |
| SLC7A11 | AL512603.2 | 0.44573924 | 1.67E-21 | postive |
| TLN1 | AL512603.2 | 0.411089381 | 3.13E-18 | postive |
| NUBPL | PAXIP1-AS2 | 0.417135346 | 8.93E-19 | postive |
| NCKAP1 | PAXIP1-AS2 | 0.403340836 | 1.50E-17 | postive |
| CD2AP | PAXIP1-AS2 | 0.495408307 | 6.69E-27 | postive |
| CD2AP | AC092802.1 | 0.455621715 | 1.65E-22 | postive |
| CD2AP | AL021707.4 | 0.470154373 | 4.78E-24 | postive |
| NUBPL | AL157400.4 | 0.417274614 | 8.67E-19 | postive |
| CD2AP | AC099482.1 | 0.425688059 | 1.45E-19 | postive |
| CD2AP | NCBP2-AS1 | 0.492364292 | 1.52E-26 | postive |
| SLC7A11 | AC110609.1 | 0.499534205 | 2.17E-27 | postive |
| CD2AP | AP005432.1 | 0.438080345 | 9.51E-21 | postive |
| CD2AP | AC026803.3 | 0.493180579 | 1.22E-26 | postive |
| MYH10 | DNM3OS | 0.547381091 | 1.40E-33 | postive |
| CD2AP | AC068790.2 | 0.428456792 | 7.97E-20 | postive |
| SLC7A11 | AC092171.1 | 0.448399331 | 9.01E-22 | postive |
| DSTN | AC079313.2 | 0.617853521 | 9.83E-45 | postive |
| FLNA | AC079313.2 | 0.681796032 | 1.25E-57 | postive |
| TLN1 | AC079313.2 | 0.454508621 | 2.15E-22 | postive |
| ACTN1 | AC079313.2 | 0.492048565 | 1.66E-26 | postive |
| SLC7A11 | AC011510.1 | 0.664618178 | 7.52E-54 | postive |
| CD2AP | AC015849.3 | 0.415798653 | 1.18E-18 | postive |
| ACTB | GORAB-AS1 | 0.440799209 | 5.15E-21 | postive |
| ACTN1 | GORAB-AS1 | 0.512697971 | 5.37E-29 | postive |
| ACTB | AC004148.1 | -0.414766662 | 1.46E-18 | negative |
| SLC7A11 | AL731537.1 | 0.707432146 | 8.94E-64 | postive |
| SLC7A11 | AC087286.4 | 0.426036154 | 1.35E-19 | postive |
| SLC7A11 | AC092053.3 | 0.654321854 | 1.05E-51 | postive |
| SLC7A11 | AC009054.1 | 0.429544258 | 6.29E-20 | postive |
| NUBPL | AC243654.1 | 0.40200074 | 1.96E-17 | postive |
| CD2AP | AC243654.1 | 0.409519677 | 4.31E-18 | postive |
| TLN2 | AC243654.1 | 0.428168208 | 8.49E-20 | postive |
| SLC7A11 | AC012076.1 | 0.451561261 | 4.31E-22 | postive |
| CD2AP | AC073046.3 | 0.401573706 | 2.14E-17 | postive |
| NDUFA11 | KMT2E-AS1 | 0.502157221 | 1.05E-27 | postive |
| NDUFA11 | AC124016.1 | 0.432392665 | 3.37E-20 | postive |
| ACTB | NKILA | 0.43421861 | 2.25E-20 | postive |
| ACTN1 | NKILA | 0.423277494 | 2.44E-19 | postive |
| SLC7A11 | AL445649.1 | 0.57741009 | 5.29E-38 | postive |
| DSTN | CARMN | 0.534177039 | 9.01E-32 | postive |
| FLNA | CARMN | 0.626049343 | 3.21E-46 | postive |
| TLN1 | CARMN | 0.444595822 | 2.17E-21 | postive |
| ACTN1 | CARMN | 0.43188686 | 3.77E-20 | postive |
| CD2AP | Z99127.3 | 0.405770838 | 9.22E-18 | postive |
| ACTB | AL691482.3 | -0.406244232 | 8.38E-18 | negative |
| NUBPL | AC073529.1 | 0.407609079 | 6.36E-18 | postive |
| CD2AP | AC073529.1 | 0.422784379 | 2.71E-19 | postive |
| SLC7A11 | FO680682.1 | 0.467249518 | 9.83E-24 | postive |
| NDUFA11 | AC010422.2 | 0.555037184 | 1.15E-34 | postive |
| CD2AP | AL450384.1 | 0.456628976 | 1.30E-22 | postive |
| SLC7A11 | AL596223.1 | 0.775983039 | 4.44E-84 | postive |
| FLNB | AC018755.5 | 0.419821357 | 5.08E-19 | postive |
| CD2AP | AC234775.2 | 0.422081714 | 3.14E-19 | postive |
| SLC7A11 | ANKRD44-IT1 | 0.684446095 | 3.11E-58 | postive |
| IQGAP1 | AC090587.1 | 0.48836682 | 4.42E-26 | postive |
| NDUFA11 | AC023302.1 | 0.468145122 | 7.88E-24 | postive |
| ACTN1 | LINC01705 | 0.404486355 | 1.19E-17 | postive |
| TLN2 | AP001107.9 | 0.404164299 | 1.27E-17 | postive |
| NDUFA11 | AC127024.6 | 0.446502083 | 1.40E-21 | postive |
| CD2AP | AC009163.4 | 0.424828895 | 1.75E-19 | postive |
| CD2AP | AC006017.1 | 0.436286657 | 1.42E-20 | postive |
| NUBPL | AC004943.2 | 0.432026951 | 3.65E-20 | postive |
| CD2AP | AC008870.4 | 0.450748263 | 5.21E-22 | postive |
| NDUFA11 | AL008582.1 | 0.423284608 | 2.43E-19 | postive |
| CD2AP | MCCC1-AS1 | 0.480192068 | 3.74E-25 | postive |
| FLNA | BX323043.1 | 0.434726335 | 2.01E-20 | postive |
| DSTN | ADAMTS9-AS1 | 0.602284508 | 4.98E-42 | postive |
| FLNA | ADAMTS9-AS1 | 0.620952749 | 2.73E-45 | postive |
| TLN1 | ADAMTS9-AS1 | 0.429082519 | 6.96E-20 | postive |
| NDUFA11 | AL096701.4 | 0.413684725 | 1.83E-18 | postive |
| CD2AP | AC008543.1 | 0.41098629 | 3.19E-18 | postive |
| NDUFA11 | AL158063.1 | 0.431327752 | 4.26E-20 | postive |
| NDUFA11 | AC073508.3 | 0.415732067 | 1.20E-18 | postive |
| NDUFA11 | AC020765.2 | 0.459109336 | 7.16E-23 | postive |
| CD2AP | UBOX5-AS1 | 0.408568121 | 5.23E-18 | postive |
| NDUFA11 | AC011445.1 | 0.437535076 | 1.07E-20 | postive |
| CD2AP | AC007881.4 | 0.459716195 | 6.19E-23 | postive |
| CD2AP | AC025442.2 | 0.408398327 | 5.42E-18 | postive |
| CD2AP | AC004893.2 | 0.434708398 | 2.02E-20 | postive |
| DSTN | AL162424.1 | 0.480245094 | 3.69E-25 | postive |
| FLNA | AL162424.1 | 0.621665679 | 2.03E-45 | postive |
| TLN1 | AL162424.1 | 0.415495704 | 1.26E-18 | postive |
| ACTN1 | AL162424.1 | 0.482652884 | 1.98E-25 | postive |
| CD2AP | AC087294.1 | 0.423719378 | 2.22E-19 | postive |
| CD2AP | MAL2-AS1 | 0.464610989 | 1.88E-23 | postive |
| TLN2 | GATA6-AS1 | 0.496298654 | 5.26E-27 | postive |
| CD2AP | AC023794.4 | 0.452261349 | 3.65E-22 | postive |
| CD2AP | LINC02109 | 0.41853638 | 6.65E-19 | postive |
| CD2AP | AL050309.1 | 0.469649433 | 5.42E-24 | postive |
| CD2AP | AC139792.1 | 0.429356296 | 6.55E-20 | postive |
| NDUFA11 | AL035461.2 | 0.402728019 | 1.70E-17 | postive |
| NDUFA11 | SCAT2 | 0.401899573 | 2.00E-17 | postive |
| SLC7A11 | Z82243.1 | 0.508504504 | 1.78E-28 | postive |
| SLC7A11 | AC234772.1 | 0.675761656 | 2.85E-56 | postive |
| CD2AP | LAMTOR5-AS1 | 0.429230822 | 6.74E-20 | postive |
| CD2AP | AL390719.3 | 0.461895437 | 3.65E-23 | postive |
| CD2AP | AC139887.1 | 0.446309043 | 1.46E-21 | postive |
| CD2AP | AC125494.1 | 0.454275031 | 2.27E-22 | postive |
| SLC7A11 | AC138207.7 | 0.55695799 | 6.10E-35 | postive |
| CD2AP | AL139353.2 | 0.421016555 | 3.94E-19 | postive |
| CD2AP | AC008543.3 | 0.514253795 | 3.43E-29 | postive |
| CD2AP | AC092821.3 | 0.423033425 | 2.57E-19 | postive |
| CD2AP | AP001619.1 | 0.465857164 | 1.39E-23 | postive |
| CD2AP | LINC01355 | 0.42078147 | 4.14E-19 | postive |
| CD2AP | AC005828.4 | 0.443359005 | 2.88E-21 | postive |
| SLC7A11 | AC108053.1 | 0.455721571 | 1.61E-22 | postive |
| CD2AP | AC004967.2 | 0.436886149 | 1.24E-20 | postive |
| SLC7A11 | MSC-AS1 | 0.477695734 | 7.11E-25 | postive |
| SLC7A11 | AC006160.1 | 0.581129033 | 1.39E-38 | postive |
| CD2AP | AC124069.1 | 0.43019342 | 5.46E-20 | postive |
| NDUFA11 | Z69706.1 | 0.412660919 | 2.26E-18 | postive |
| MYH10 | LINC01614 | 0.404636943 | 1.16E-17 | postive |
| ACTN1 | LINC01614 | 0.400803741 | 2.49E-17 | postive |
| CD2AP | AL590729.1 | 0.442197218 | 3.75E-21 | postive |
| SLC7A11 | AL355073.2 | 0.47596578 | 1.11E-24 | postive |
| MYH10 | AL049871.1 | 0.606447191 | 9.75E-43 | postive |
| CD2AP | AL391684.1 | 0.402965933 | 1.62E-17 | postive |
| CD2AP | PDXDC2P-NPIPB14P | 0.433708684 | 2.52E-20 | postive |
| NDUFA11 | AC074212.1 | 0.433604999 | 2.58E-20 | postive |
| MYH10 | AP005328.1 | 0.44465845 | 2.14E-21 | postive |
| FLNA | LINC02104 | 0.418514362 | 6.69E-19 | postive |
| TLN1 | LINC02104 | 0.401893042 | 2.00E-17 | postive |
| ACTN1 | LINC02104 | 0.419536358 | 5.39E-19 | postive |
| CD2AP | AP000692.1 | 0.466545566 | 1.17E-23 | postive |
| ACTB | AL731567.1 | -0.401772116 | 2.05E-17 | negative |
| CD2AP | AL731567.1 | 0.464310632 | 2.03E-23 | postive |
| CD2AP | AL138921.1 | 0.438572453 | 8.51E-21 | postive |
| DSTN | AP004609.1 | 0.561141862 | 1.50E-35 | postive |
| FLNA | AP004609.1 | 0.541192692 | 1.01E-32 | postive |
| NDUFA11 | AL139123.1 | 0.525151568 | 1.40E-30 | postive |
| SLC7A11 | AC066613.1 | 0.503403256 | 7.44E-28 | postive |
| NDUFA11 | AL023284.4 | 0.468700493 | 6.86E-24 | postive |
| CD2AP | AC025031.4 | 0.442643272 | 3.39E-21 | postive |

Correlation between lnRNAs and 27 disulfidptosis-related genes.
